# Supplementary figures and images for: Packaging style design based on visual semantic segmentation technology and intelligent cyber physical system (part 2 of 3)
Source: PeerJ Comput Sci. 2023 Jul 10;9:e1451. doi: 10.7717/peerj-cs.1451 (PMC10403159; doi:10.7717/peerj-cs.1451)

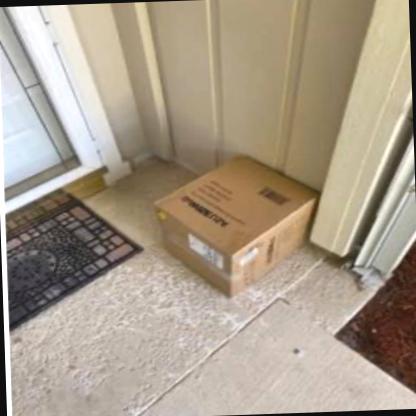

Supplement: Supplemental Information 1 [file peerj-cs-09-1451-s001.zip › train/7_jpg.rf.9bc406fd30e4d8e5a2824b29f5ebd007.jpg]

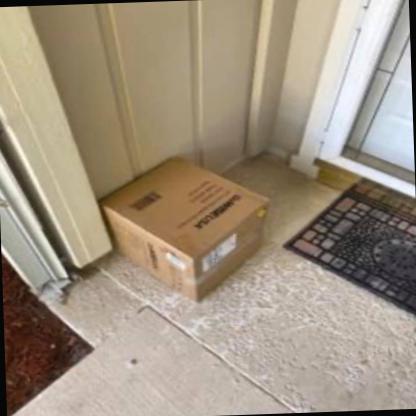

Supplement: Supplemental Information 1 [file peerj-cs-09-1451-s001.zip › train/7_jpg.rf.ee1e513903f5cb2ff0be0a11cfc236f9.jpg]

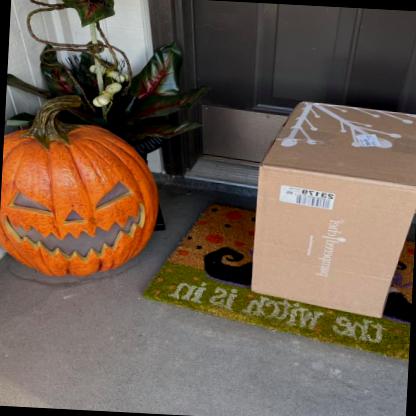

Supplement: Supplemental Information 1 [file peerj-cs-09-1451-s001.zip › train/8_jpg.rf.39d097ce9d72ac95cdd9fc506f4af99f.jpg]

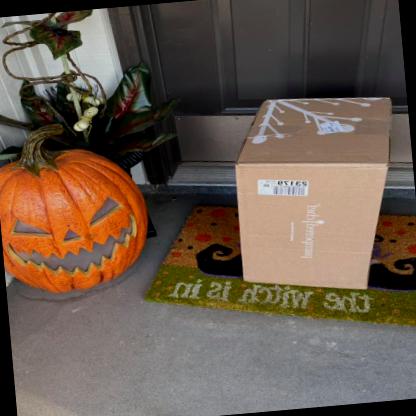

Supplement: Supplemental Information 1 [file peerj-cs-09-1451-s001.zip › train/8_jpg.rf.b48feb17fe048bd1f21fcd8e887ee53b.jpg]

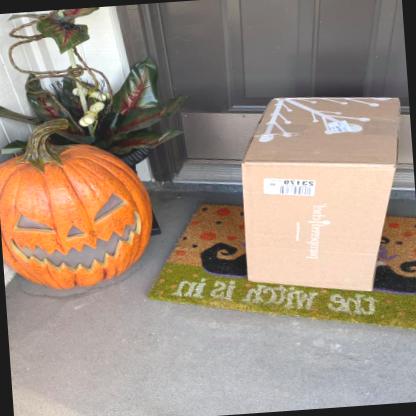

Supplement: Supplemental Information 1 [file peerj-cs-09-1451-s001.zip › train/8_jpg.rf.d0b5d595a5a239fd8a2824784c8bde32.jpg]

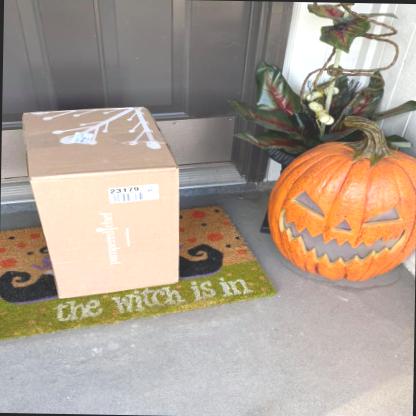

Supplement: Supplemental Information 1 [file peerj-cs-09-1451-s001.zip › train/8_jpg.rf.d9f5f3033c8b605299d71d718c42cb47.jpg]

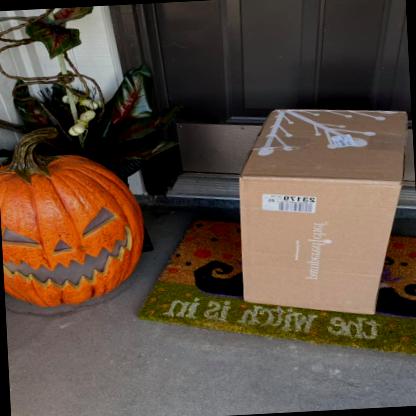

Supplement: Supplemental Information 1 [file peerj-cs-09-1451-s001.zip › train/8_jpg.rf.de7720dd6dbece3fd7bd5c83a9a6109c.jpg]

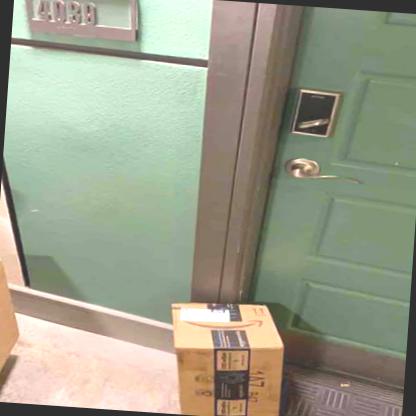

Supplement: Supplemental Information 1 [file peerj-cs-09-1451-s001.zip › train/9_jpg.rf.2b5aacc2fc51aa54ae9cc598c6101000.jpg]

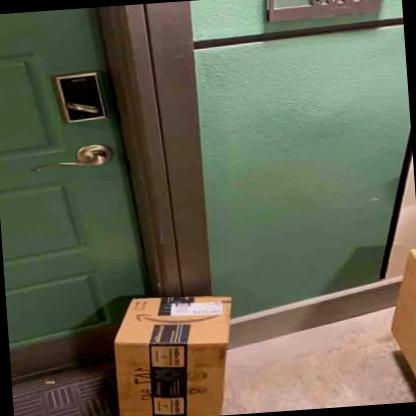

Supplement: Supplemental Information 1 [file peerj-cs-09-1451-s001.zip › train/9_jpg.rf.531690aea3a42a46eca9ddfa875bb6e2.jpg]

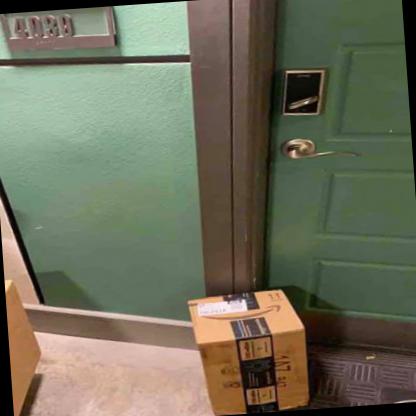

Supplement: Supplemental Information 1 [file peerj-cs-09-1451-s001.zip › train/9_jpg.rf.5b96fb3b7d62f0d6b3f874cbdc7c7e59.jpg]

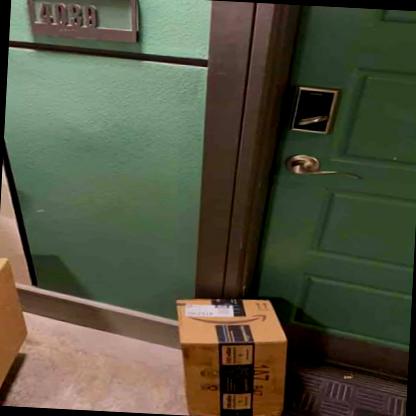

Supplement: Supplemental Information 1 [file peerj-cs-09-1451-s001.zip › train/9_jpg.rf.71a9cd6877cad493510f8d77f5228aea.jpg]

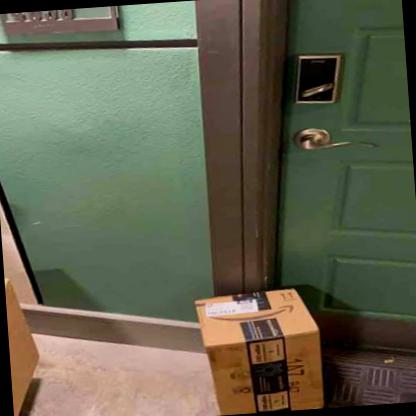

Supplement: Supplemental Information 1 [file peerj-cs-09-1451-s001.zip › train/9_jpg.rf.92fa2285fa3933daf0153602cb2b6c7a.jpg]

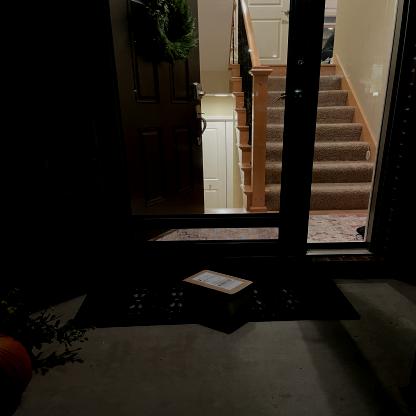

Supplement: Supplemental Information 1 [file peerj-cs-09-1451-s001.zip › train/IMG_3238_jpeg.rf.62fcda3178ff26467074c42b6146a47b.jpg]

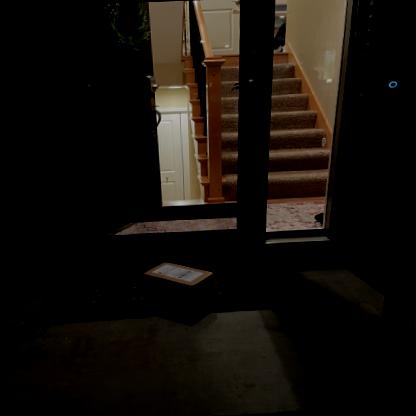

Supplement: Supplemental Information 1 [file peerj-cs-09-1451-s001.zip › train/IMG_3238_jpeg.rf.935d029214e52509809646293ad78b80.jpg]

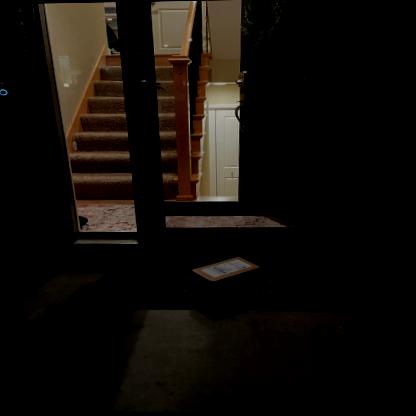

Supplement: Supplemental Information 1 [file peerj-cs-09-1451-s001.zip › train/IMG_3238_jpeg.rf.ce2c60826cc8da80ed91f3155444cbb6.jpg]

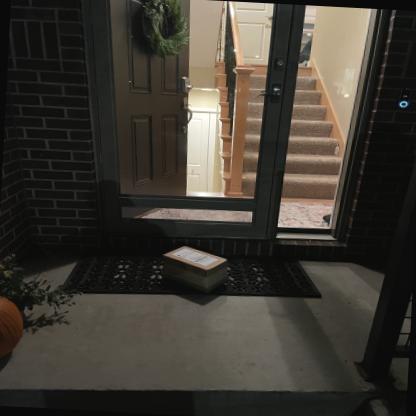

Supplement: Supplemental Information 1 [file peerj-cs-09-1451-s001.zip › train/IMG_3238_jpeg.rf.cfde39a66ffdec891c2e79483672ada9.jpg]

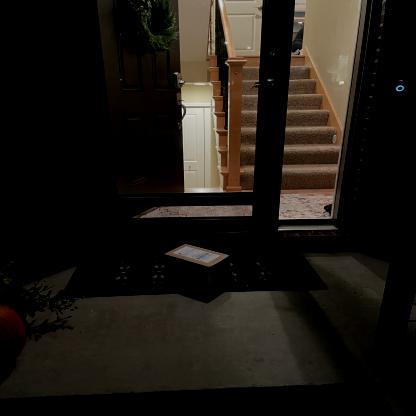

Supplement: Supplemental Information 1 [file peerj-cs-09-1451-s001.zip › train/IMG_3238_jpeg.rf.fc25f32794aa6ce8cdbfb887c9341c92.jpg]

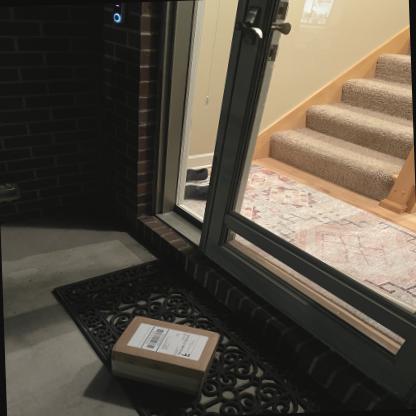

Supplement: Supplemental Information 1 [file peerj-cs-09-1451-s001.zip › train/IMG_3239_jpeg.rf.2299d70498f75eac0518c7e0b29b911e.jpg]

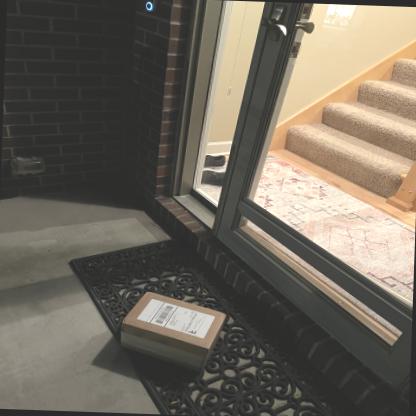

Supplement: Supplemental Information 1 [file peerj-cs-09-1451-s001.zip › train/IMG_3239_jpeg.rf.6feae1499908daa5ab5f2c9dda3860dd.jpg]

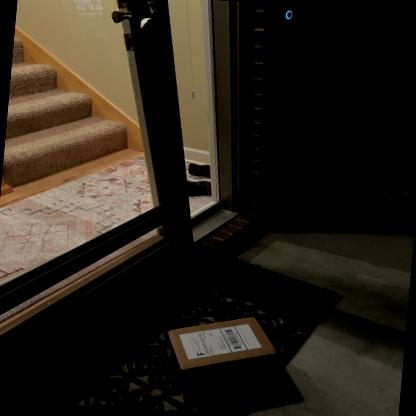

Supplement: Supplemental Information 1 [file peerj-cs-09-1451-s001.zip › train/IMG_3239_jpeg.rf.ae18333a2ecd2b662e0efc135747ba54.jpg]

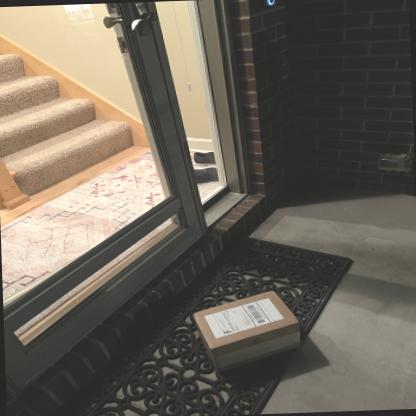

Supplement: Supplemental Information 1 [file peerj-cs-09-1451-s001.zip › train/IMG_3239_jpeg.rf.c37bff39e5dcee0f3a7aff263c810d92.jpg]

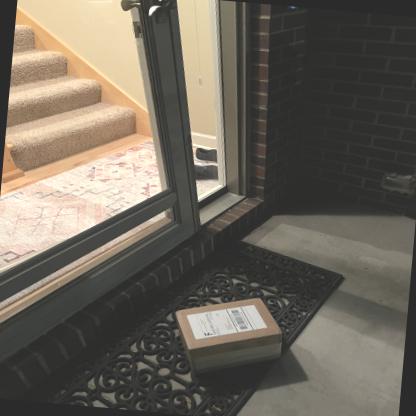

Supplement: Supplemental Information 1 [file peerj-cs-09-1451-s001.zip › train/IMG_3239_jpeg.rf.ecf02937587224d49ac751c6fe383bd6.jpg]

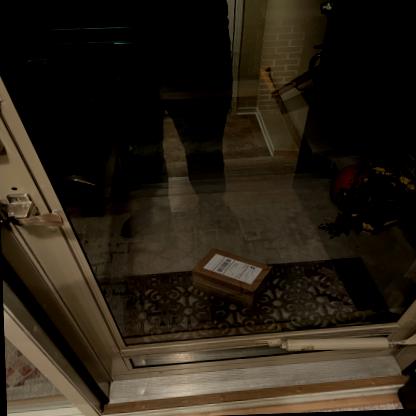

Supplement: Supplemental Information 1 [file peerj-cs-09-1451-s001.zip › train/IMG_3240_jpeg.rf.3052a8f7a96311fb446187e264cba252.jpg]

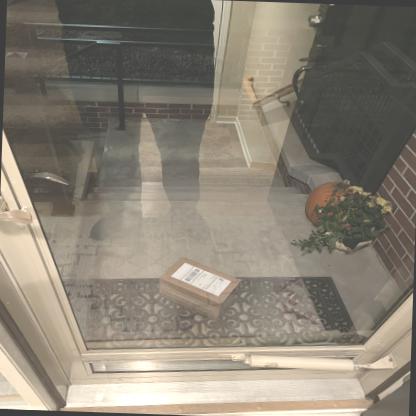

Supplement: Supplemental Information 1 [file peerj-cs-09-1451-s001.zip › train/IMG_3240_jpeg.rf.46074417bd6ccc076c738c8db4a29e99.jpg]

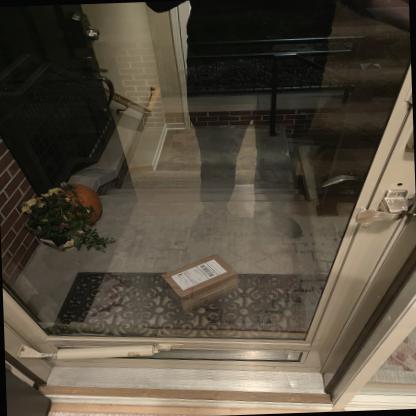

Supplement: Supplemental Information 1 [file peerj-cs-09-1451-s001.zip › train/IMG_3240_jpeg.rf.979a62e74b04de20eeefde5230a887f0.jpg]

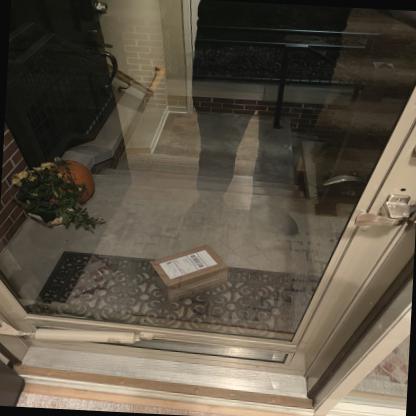

Supplement: Supplemental Information 1 [file peerj-cs-09-1451-s001.zip › train/IMG_3240_jpeg.rf.ae8b3c3a714020b39ee52ced0c8a1560.jpg]

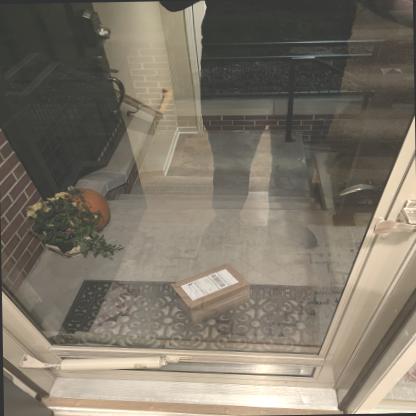

Supplement: Supplemental Information 1 [file peerj-cs-09-1451-s001.zip › train/IMG_3240_jpeg.rf.e405f1eb08b6e11f23d0ffb24d679874.jpg]

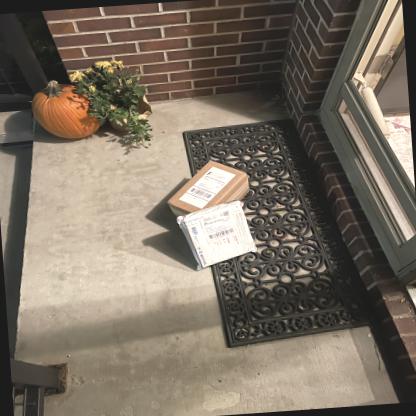

Supplement: Supplemental Information 1 [file peerj-cs-09-1451-s001.zip › train/IMG_3242_jpeg.rf.1084b70673d81e6e24f58776afebe782.jpg]

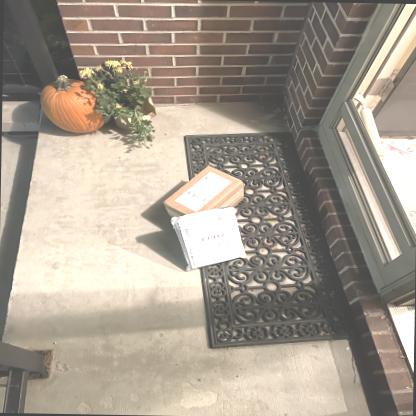

Supplement: Supplemental Information 1 [file peerj-cs-09-1451-s001.zip › train/IMG_3242_jpeg.rf.509244b9b929ee12f4cb6b0c5252e378.jpg]

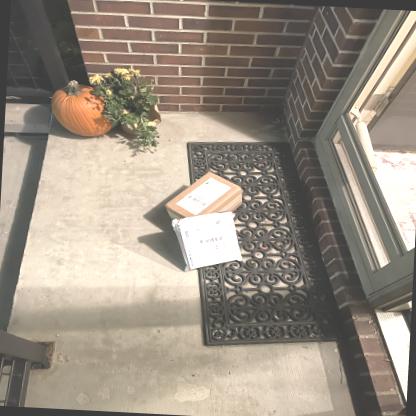

Supplement: Supplemental Information 1 [file peerj-cs-09-1451-s001.zip › train/IMG_3242_jpeg.rf.81ed0e952deb2ef574128c694a2b3124.jpg]

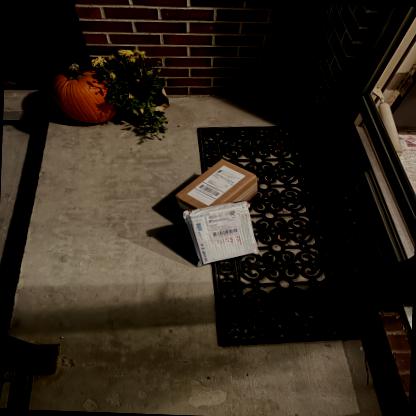

Supplement: Supplemental Information 1 [file peerj-cs-09-1451-s001.zip › train/IMG_3242_jpeg.rf.98a16de977976bb734d46c8b371e2a77.jpg]

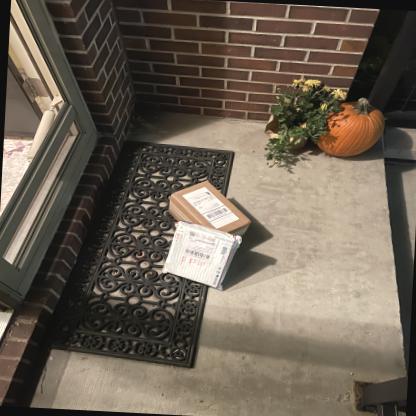

Supplement: Supplemental Information 1 [file peerj-cs-09-1451-s001.zip › train/IMG_3242_jpeg.rf.e3b3ee0560d4414f7c7052823319c7d8.jpg]

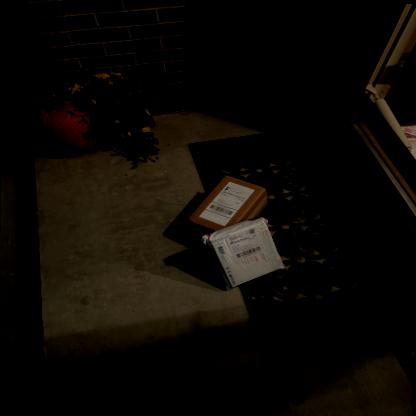

Supplement: Supplemental Information 1 [file peerj-cs-09-1451-s001.zip › train/IMG_3243_jpeg.rf.0445b762c5ed798313fa58fcb176503e.jpg]

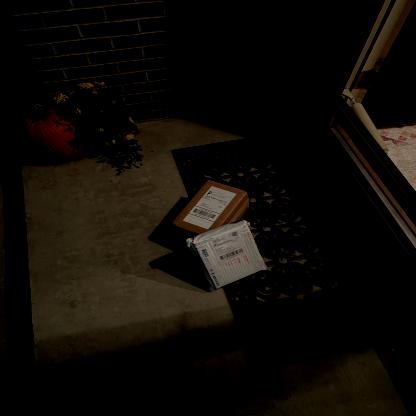

Supplement: Supplemental Information 1 [file peerj-cs-09-1451-s001.zip › train/IMG_3243_jpeg.rf.2e4900b2ed7998714fd47005ee7fd320.jpg]

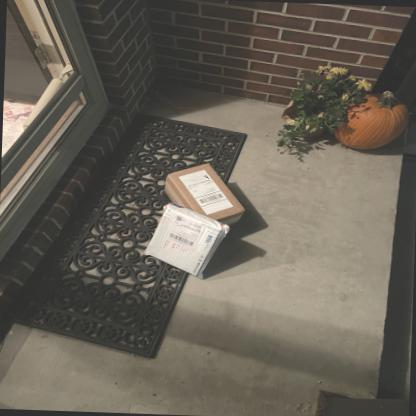

Supplement: Supplemental Information 1 [file peerj-cs-09-1451-s001.zip › train/IMG_3243_jpeg.rf.31ead35defcbf0d34a76c8cc4b064de6.jpg]

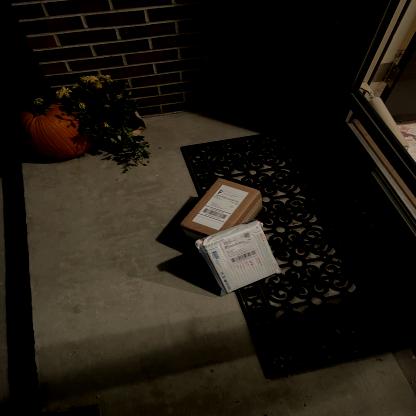

Supplement: Supplemental Information 1 [file peerj-cs-09-1451-s001.zip › train/IMG_3243_jpeg.rf.9b2c4a7b551f7a2ea60052107a2f8aea.jpg]

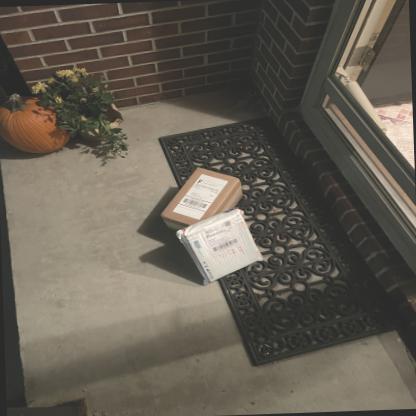

Supplement: Supplemental Information 1 [file peerj-cs-09-1451-s001.zip › train/IMG_3243_jpeg.rf.c55998fcab234a6b5c6ab63c06e9a1e2.jpg]

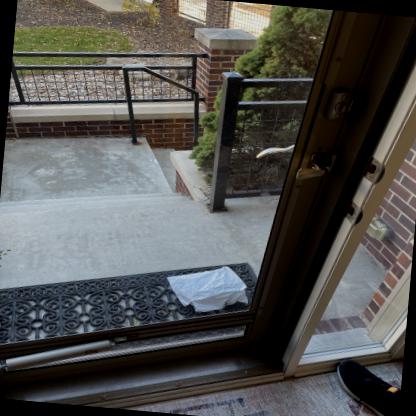

Supplement: Supplemental Information 1 [file peerj-cs-09-1451-s001.zip › train/IMG_3248_jpeg.rf.6313f8de49c5e0dea1b861c7f7c72616.jpg]

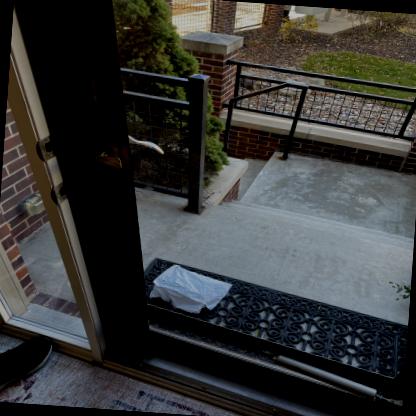

Supplement: Supplemental Information 1 [file peerj-cs-09-1451-s001.zip › train/IMG_3248_jpeg.rf.6922c7fafbfa07db6c54b0c56bf63844.jpg]

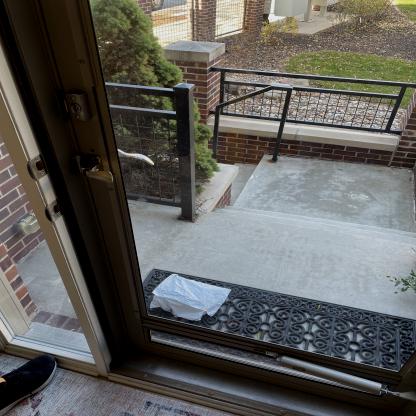

Supplement: Supplemental Information 1 [file peerj-cs-09-1451-s001.zip › train/IMG_3248_jpeg.rf.a0716b362ef186e243c9c4d316f1dcca.jpg]

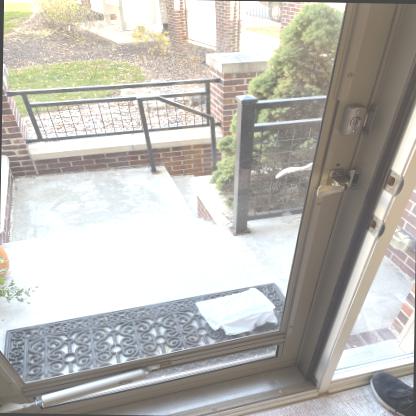

Supplement: Supplemental Information 1 [file peerj-cs-09-1451-s001.zip › train/IMG_3248_jpeg.rf.c4909ac2f3e1a7f55cde97eecba810ec.jpg]

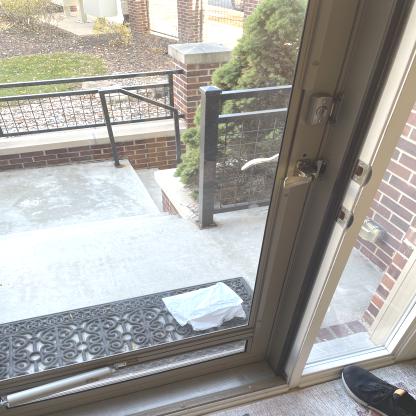

Supplement: Supplemental Information 1 [file peerj-cs-09-1451-s001.zip › train/IMG_3248_jpeg.rf.e0db1eb41934a39eba520e4f56e21a13.jpg]

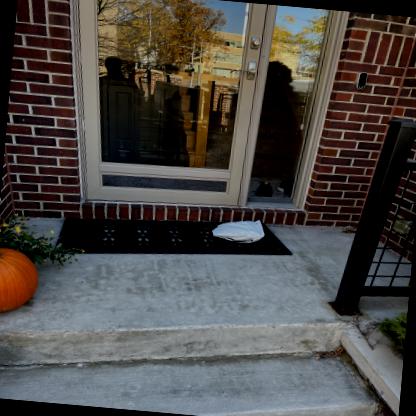

Supplement: Supplemental Information 1 [file peerj-cs-09-1451-s001.zip › train/IMG_3249_jpeg.rf.187a44d20a1aa48a8f98860d9be85e17.jpg]

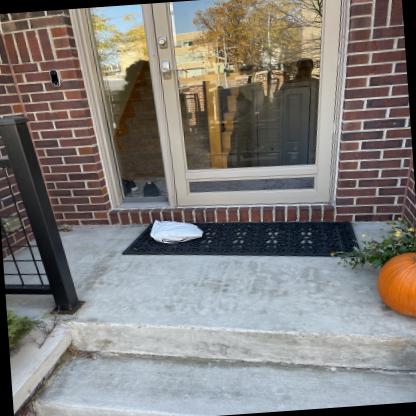

Supplement: Supplemental Information 1 [file peerj-cs-09-1451-s001.zip › train/IMG_3249_jpeg.rf.5983c9386a3173a6948b285564c224b7.jpg]

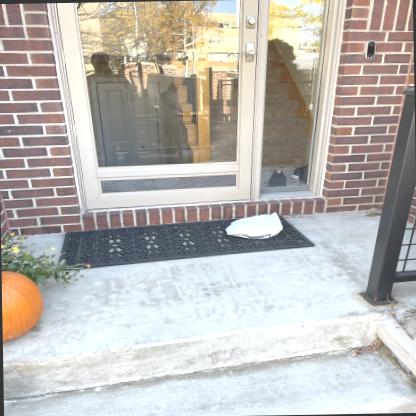

Supplement: Supplemental Information 1 [file peerj-cs-09-1451-s001.zip › train/IMG_3249_jpeg.rf.8b21dbdb932dae3923abba20a07e9fe4.jpg]

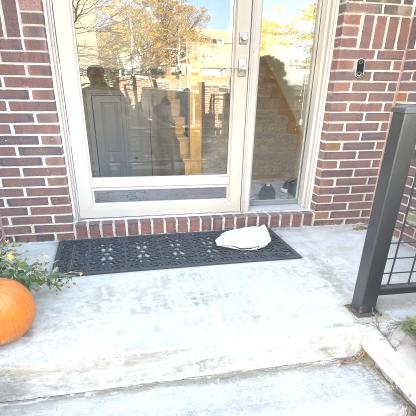

Supplement: Supplemental Information 1 [file peerj-cs-09-1451-s001.zip › train/IMG_3249_jpeg.rf.9c24c7262d87c2ec8dd43a0cbec7a178.jpg]

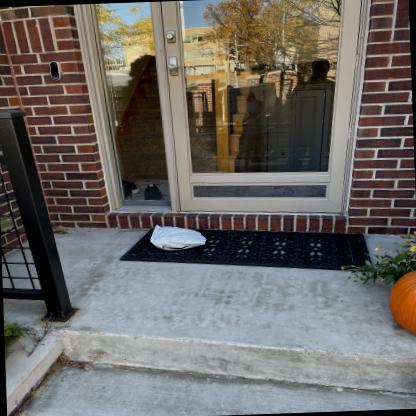

Supplement: Supplemental Information 1 [file peerj-cs-09-1451-s001.zip › train/IMG_3249_jpeg.rf.cd173a6d5ce093422fbd1740b6dbd3b2.jpg]

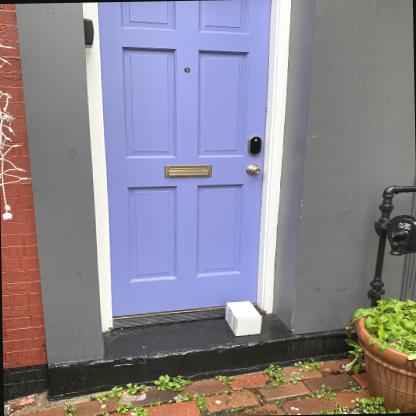

Supplement: Supplemental Information 1 [file peerj-cs-09-1451-s001.zip › train/IMG_6817_jpg.rf.2b27ee700087707e9721d9ef99180953.jpg]

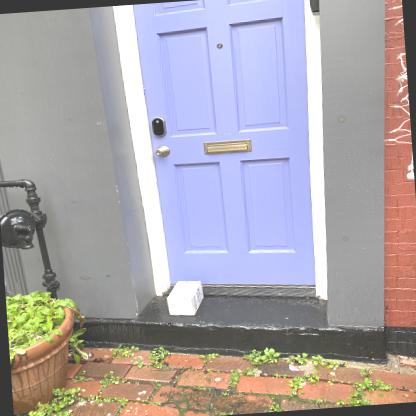

Supplement: Supplemental Information 1 [file peerj-cs-09-1451-s001.zip › train/IMG_6817_jpg.rf.4f0c645ca6bfa29783ebafc3cf6ee1c0.jpg]

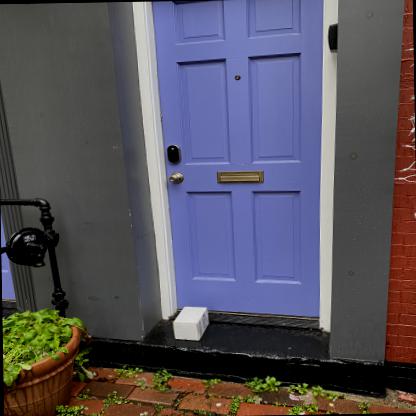

Supplement: Supplemental Information 1 [file peerj-cs-09-1451-s001.zip › train/IMG_6817_jpg.rf.570b3cee12be74810ef82d2e0cb2cc8a.jpg]

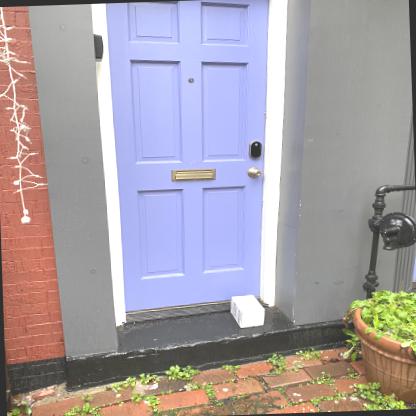

Supplement: Supplemental Information 1 [file peerj-cs-09-1451-s001.zip › train/IMG_6817_jpg.rf.844adc441aed7f7c029881844b9638e6.jpg]

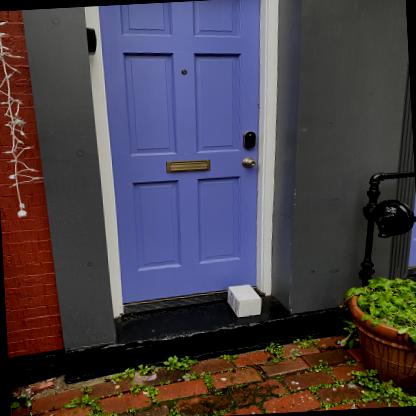

Supplement: Supplemental Information 1 [file peerj-cs-09-1451-s001.zip › train/IMG_6817_jpg.rf.bfe8f41397cf45973b1e132292980281.jpg]

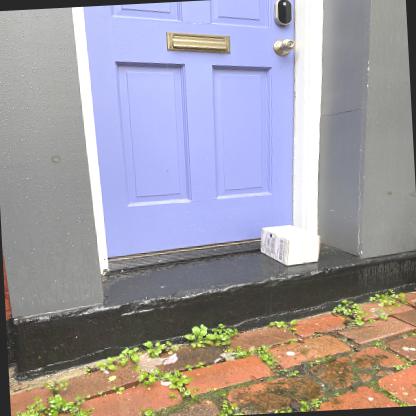

Supplement: Supplemental Information 1 [file peerj-cs-09-1451-s001.zip › train/IMG_6818_jpg.rf.4cf2cbc673b8173c113825f52ed30a16.jpg]

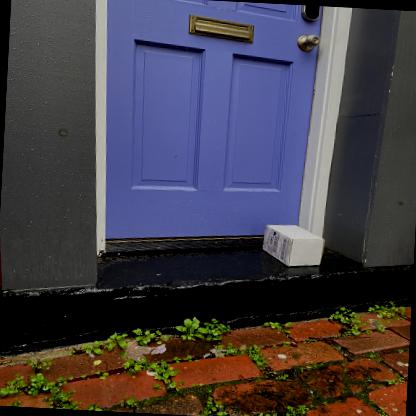

Supplement: Supplemental Information 1 [file peerj-cs-09-1451-s001.zip › train/IMG_6818_jpg.rf.538e298294e63232e75784890e05b146.jpg]

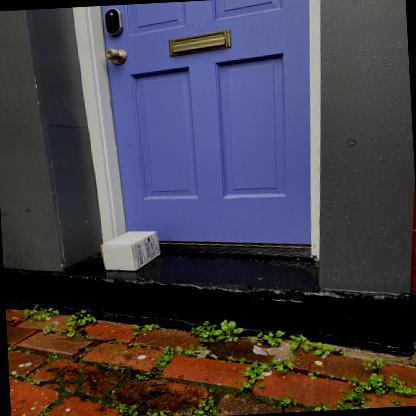

Supplement: Supplemental Information 1 [file peerj-cs-09-1451-s001.zip › train/IMG_6818_jpg.rf.88e460792f0c2f00c939494186207e02.jpg]

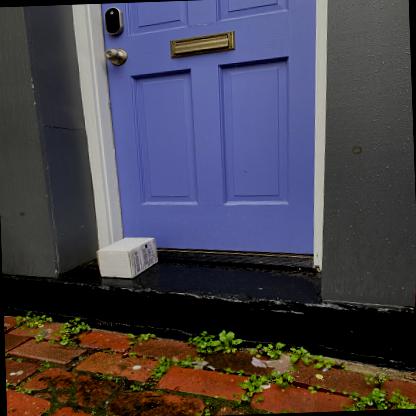

Supplement: Supplemental Information 1 [file peerj-cs-09-1451-s001.zip › train/IMG_6818_jpg.rf.d1c0488ba7217f2e3f22985453962478.jpg]

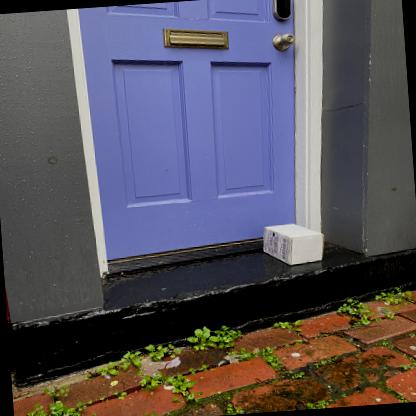

Supplement: Supplemental Information 1 [file peerj-cs-09-1451-s001.zip › train/IMG_6818_jpg.rf.f641fee3d1cb09c8b538e0f32ae5cf46.jpg]

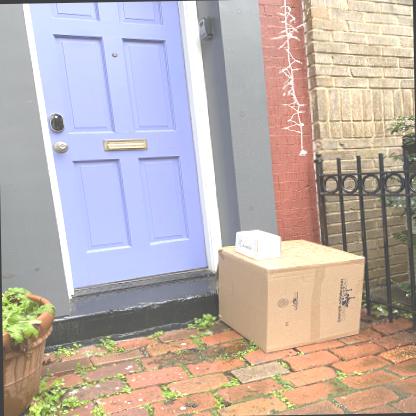

Supplement: Supplemental Information 1 [file peerj-cs-09-1451-s001.zip › train/IMG_6821_jpg.rf.085db99cf51349548087fa629a15868d.jpg]

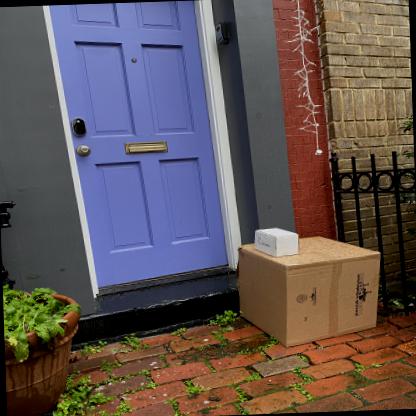

Supplement: Supplemental Information 1 [file peerj-cs-09-1451-s001.zip › train/IMG_6821_jpg.rf.5ca1f8dc4beb0175886056ee64eaf80a.jpg]

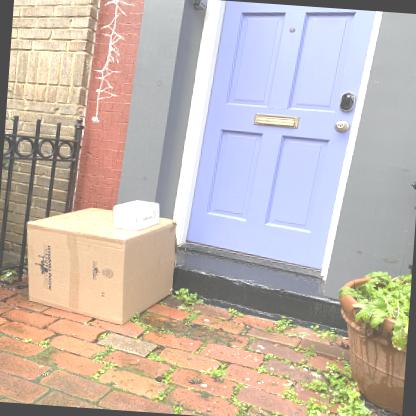

Supplement: Supplemental Information 1 [file peerj-cs-09-1451-s001.zip › train/IMG_6821_jpg.rf.7b68785404b930c5d830840dfde62f56.jpg]

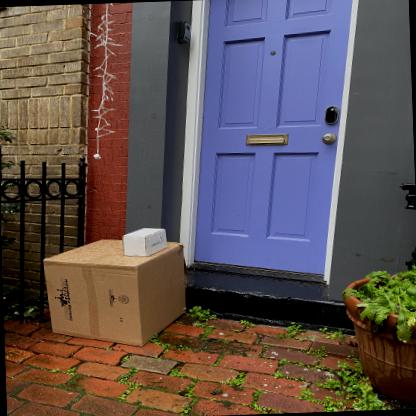

Supplement: Supplemental Information 1 [file peerj-cs-09-1451-s001.zip › train/IMG_6821_jpg.rf.826f645c8e16d9e706f2f128802a1ec8.jpg]

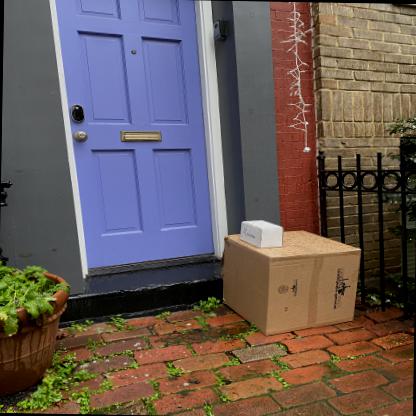

Supplement: Supplemental Information 1 [file peerj-cs-09-1451-s001.zip › train/IMG_6821_jpg.rf.c3eb2feda02ef6b66d055ed3a1b35c99.jpg]

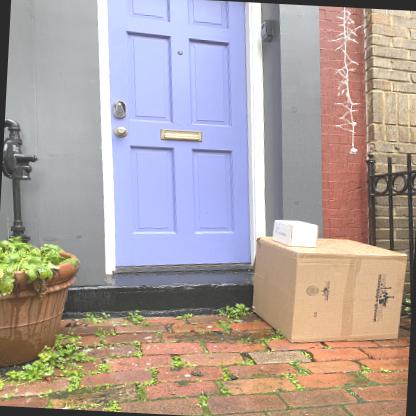

Supplement: Supplemental Information 1 [file peerj-cs-09-1451-s001.zip › train/IMG_6822_jpg.rf.1ec2110a68636d47271f185cc17c6477.jpg]

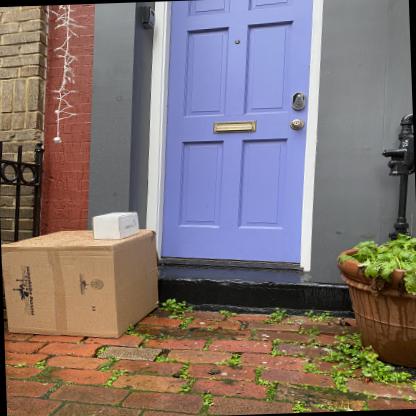

Supplement: Supplemental Information 1 [file peerj-cs-09-1451-s001.zip › train/IMG_6822_jpg.rf.8008612b9801f9dfd8829f48c3e6993b.jpg]

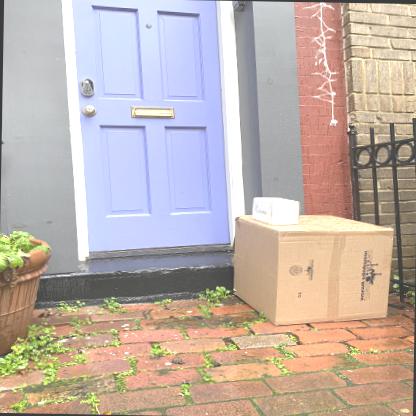

Supplement: Supplemental Information 1 [file peerj-cs-09-1451-s001.zip › train/IMG_6822_jpg.rf.8dc603ee40103634eb1201a1d66f89aa.jpg]

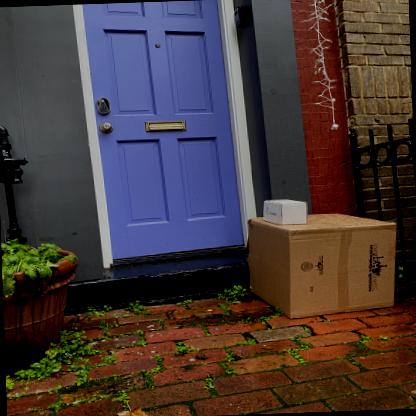

Supplement: Supplemental Information 1 [file peerj-cs-09-1451-s001.zip › train/IMG_6822_jpg.rf.a54c178d419dce1eb59b2519ec16de73.jpg]

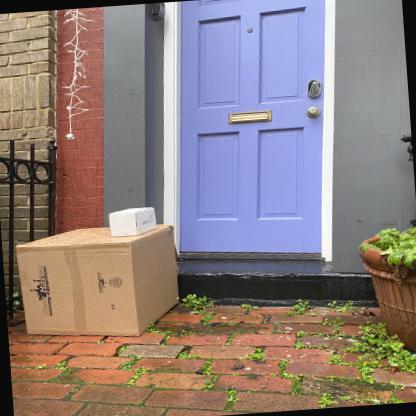

Supplement: Supplemental Information 1 [file peerj-cs-09-1451-s001.zip › train/IMG_6822_jpg.rf.bb89cc1b4a71cdaa33fbe9fb52844afd.jpg]

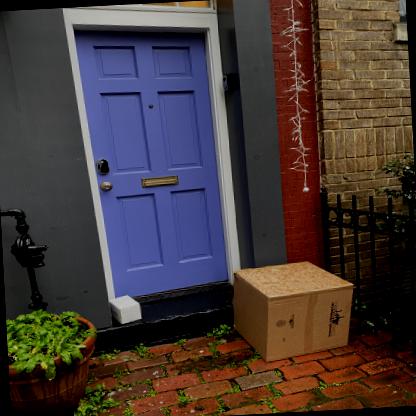

Supplement: Supplemental Information 1 [file peerj-cs-09-1451-s001.zip › train/IMG_6823_jpg.rf.0152dabc07ab1fa867917ee49b39bcee.jpg]

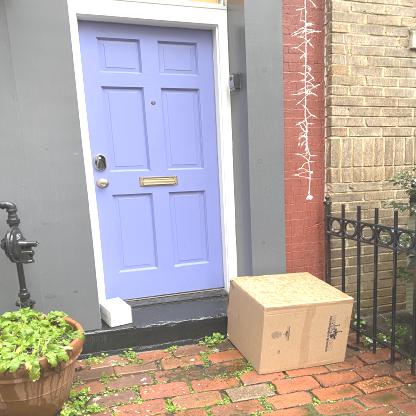

Supplement: Supplemental Information 1 [file peerj-cs-09-1451-s001.zip › train/IMG_6823_jpg.rf.23b5163605246af7d004f7438aba17f5.jpg]

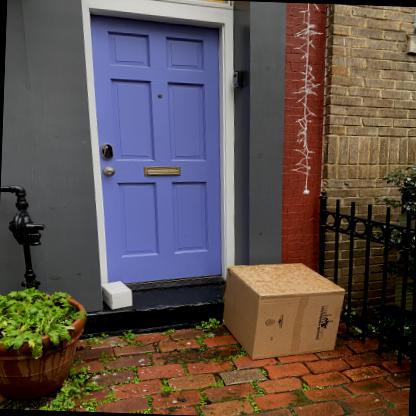

Supplement: Supplemental Information 1 [file peerj-cs-09-1451-s001.zip › train/IMG_6823_jpg.rf.28533445ef71ad35b6ac5a8837bcb80a.jpg]

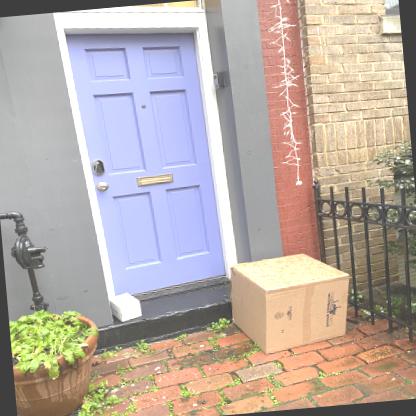

Supplement: Supplemental Information 1 [file peerj-cs-09-1451-s001.zip › train/IMG_6823_jpg.rf.552a6bff2aaef726d213786fbb747a30.jpg]

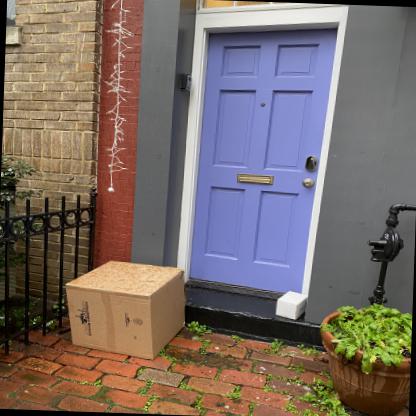

Supplement: Supplemental Information 1 [file peerj-cs-09-1451-s001.zip › train/IMG_6823_jpg.rf.699339f729b7c3406069ab56fb3eaf1c.jpg]

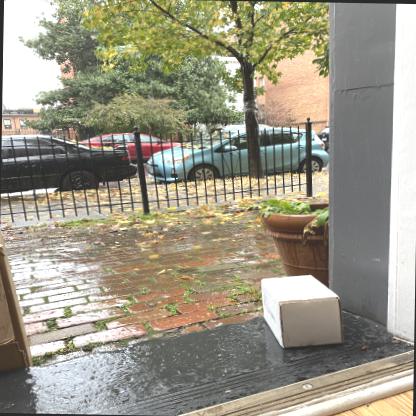

Supplement: Supplemental Information 1 [file peerj-cs-09-1451-s001.zip › train/IMG_6824_jpg.rf.10fff1daf67da29ef914c7fc85765d0d.jpg]

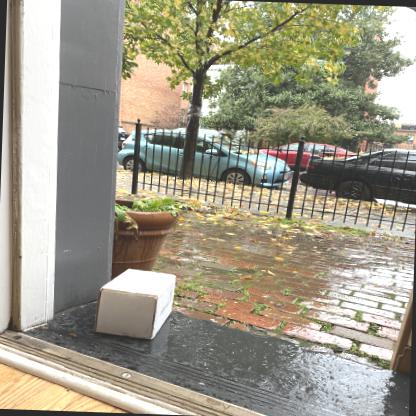

Supplement: Supplemental Information 1 [file peerj-cs-09-1451-s001.zip › train/IMG_6824_jpg.rf.4b29a32115559fd73915425f25feb992.jpg]

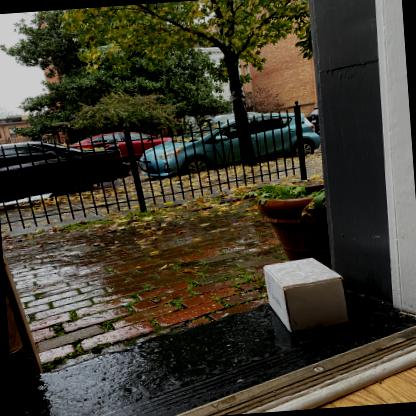

Supplement: Supplemental Information 1 [file peerj-cs-09-1451-s001.zip › train/IMG_6824_jpg.rf.503fab1e5ae74ba26fca797c54345298.jpg]

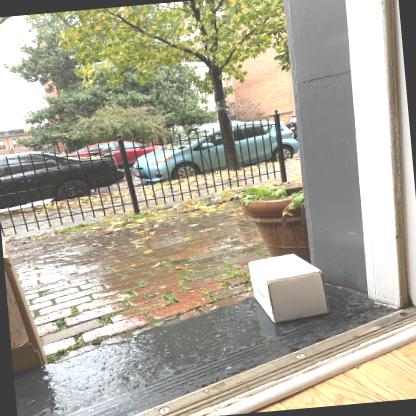

Supplement: Supplemental Information 1 [file peerj-cs-09-1451-s001.zip › train/IMG_6824_jpg.rf.d334ecdd00d1c88d227cb209eebb96dc.jpg]

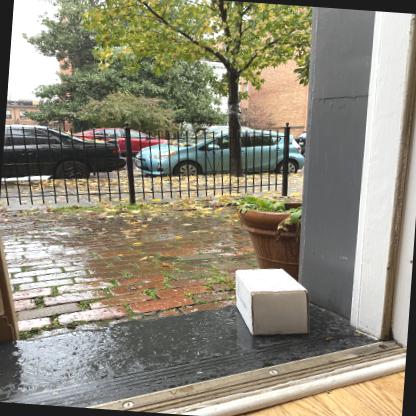

Supplement: Supplemental Information 1 [file peerj-cs-09-1451-s001.zip › train/IMG_6824_jpg.rf.e1112765afeb035d826294aedf6b09bf.jpg]

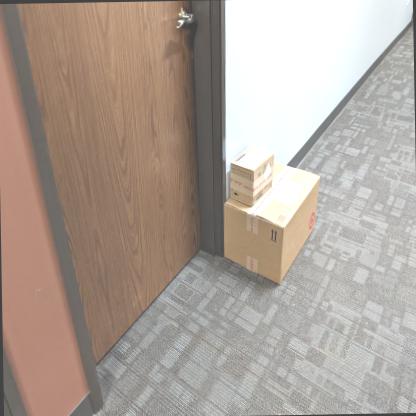

Supplement: Supplemental Information 1 [file peerj-cs-09-1451-s001.zip › train/PXL_20201101_154113387_jpg.rf.309cd309bbda8ce9323a078a80791019.jpg]

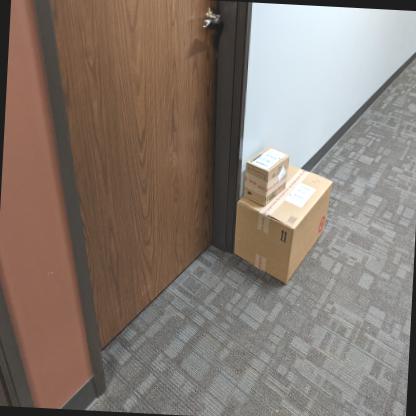

Supplement: Supplemental Information 1 [file peerj-cs-09-1451-s001.zip › train/PXL_20201101_154113387_jpg.rf.5986f049935f2858946577b2c7c6fa7e.jpg]

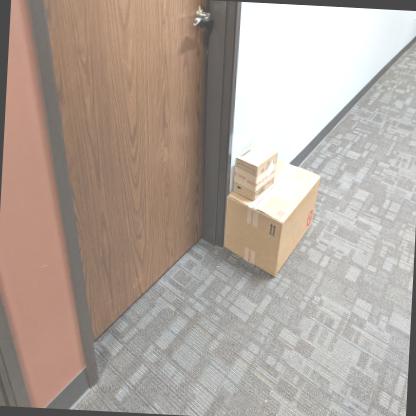

Supplement: Supplemental Information 1 [file peerj-cs-09-1451-s001.zip › train/PXL_20201101_154113387_jpg.rf.aaa8c7ebf3207fa89d89ad5969fd8451.jpg]

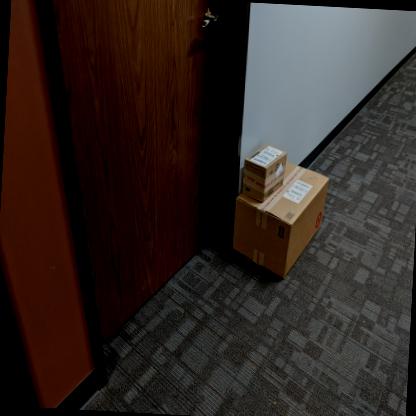

Supplement: Supplemental Information 1 [file peerj-cs-09-1451-s001.zip › train/PXL_20201101_154113387_jpg.rf.aad9f91d05c982828bdee2bec8459e7e.jpg]

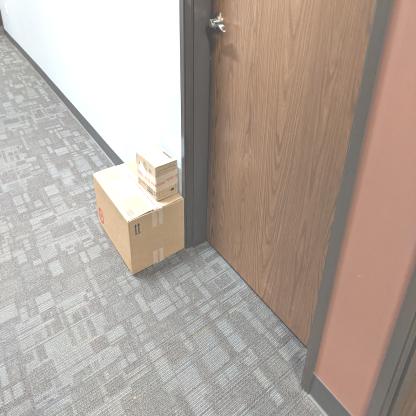

Supplement: Supplemental Information 1 [file peerj-cs-09-1451-s001.zip › train/PXL_20201101_154113387_jpg.rf.bde4683c4883388ba8999bd022227cef.jpg]

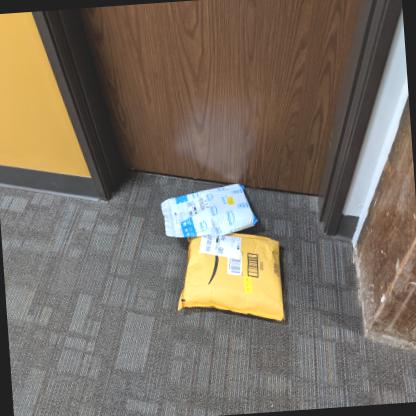

Supplement: Supplemental Information 1 [file peerj-cs-09-1451-s001.zip › train/PXL_20201103_181906837_jpg.rf.16facb85027821d1c4396e0b0c4464a8.jpg]

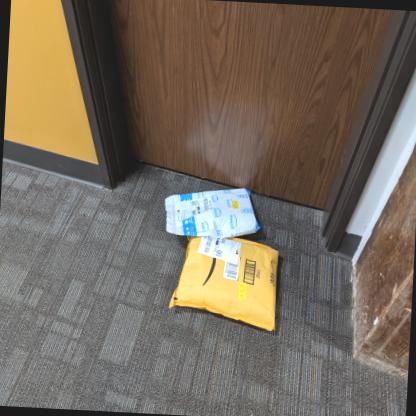

Supplement: Supplemental Information 1 [file peerj-cs-09-1451-s001.zip › train/PXL_20201103_181906837_jpg.rf.35d6cc0732355447142f1d79f24fe5d6.jpg]

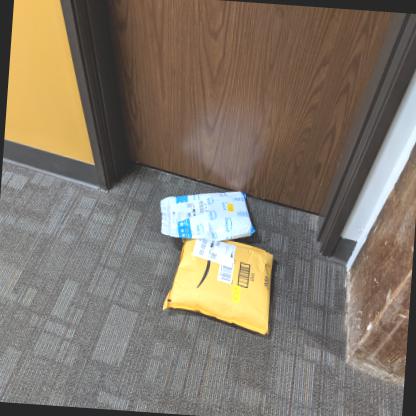

Supplement: Supplemental Information 1 [file peerj-cs-09-1451-s001.zip › train/PXL_20201103_181906837_jpg.rf.749503f526856a035fe2f434ced8896e.jpg]

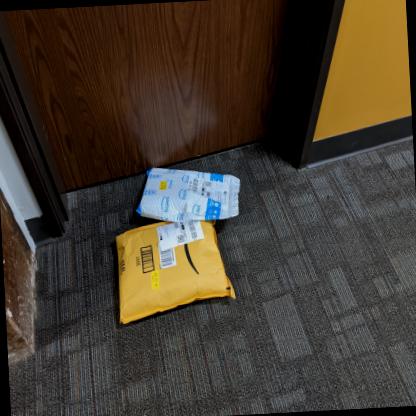

Supplement: Supplemental Information 1 [file peerj-cs-09-1451-s001.zip › train/PXL_20201103_181906837_jpg.rf.942c8fcd2ddb2d1215ce363d9b9e5f0b.jpg]

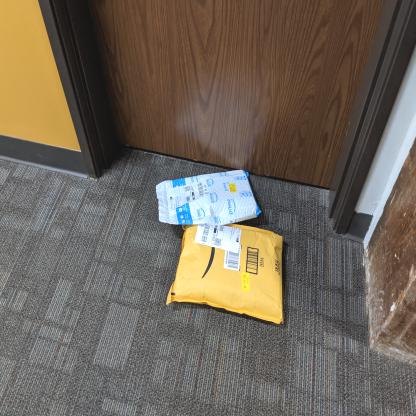

Supplement: Supplemental Information 1 [file peerj-cs-09-1451-s001.zip › train/PXL_20201103_181906837_jpg.rf.bbd8b8a92c323d90e0e815dd3a8246f9.jpg]

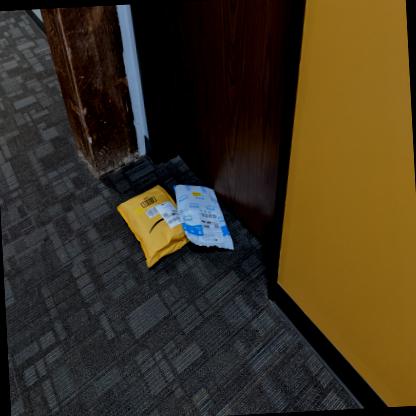

Supplement: Supplemental Information 1 [file peerj-cs-09-1451-s001.zip › train/PXL_20201103_181911592_jpg.rf.277c5b0bdaaf09ec258953dfd19df8a5.jpg]

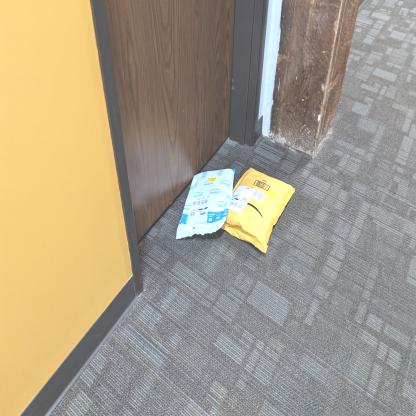

Supplement: Supplemental Information 1 [file peerj-cs-09-1451-s001.zip › train/PXL_20201103_181911592_jpg.rf.9c27d12c160121f3d51700fa27cae020.jpg]

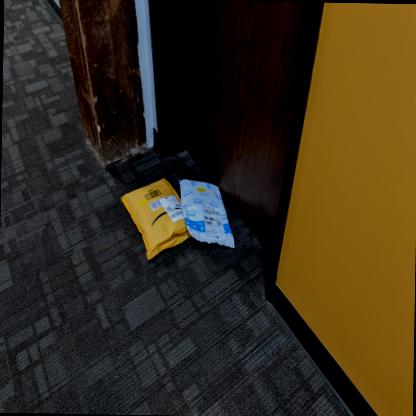

Supplement: Supplemental Information 1 [file peerj-cs-09-1451-s001.zip › train/PXL_20201103_181911592_jpg.rf.df400ec758dc388b2a1ccdae895e3b50.jpg]

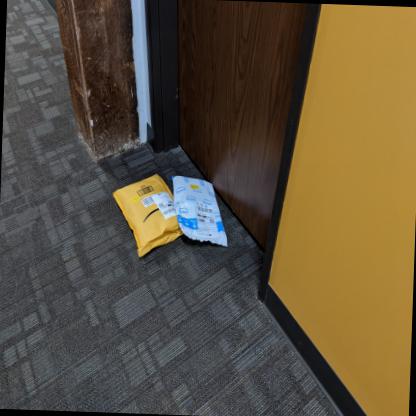

Supplement: Supplemental Information 1 [file peerj-cs-09-1451-s001.zip › train/PXL_20201103_181911592_jpg.rf.f0dcc410046e7493547a62e0651e7dd1.jpg]

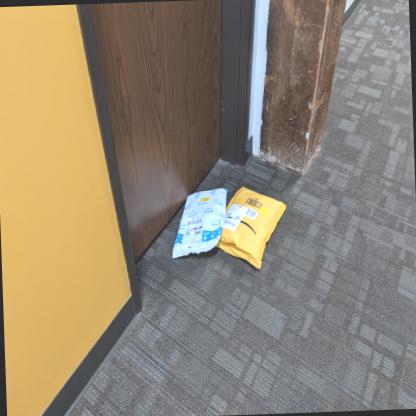

Supplement: Supplemental Information 1 [file peerj-cs-09-1451-s001.zip › train/PXL_20201103_181911592_jpg.rf.ff9af091a355f1a1bfb5d6690c9394e8.jpg]

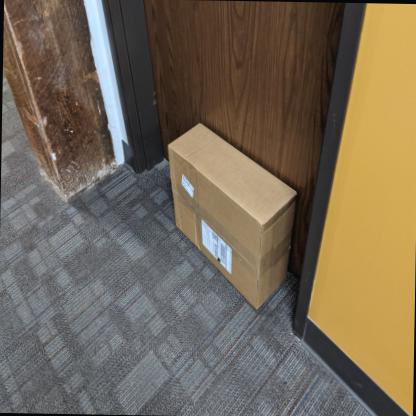

Supplement: Supplemental Information 1 [file peerj-cs-09-1451-s001.zip › train/PXL_20201105_173148569_jpg.rf.22a045bf175813f2b27901439111e525.jpg]

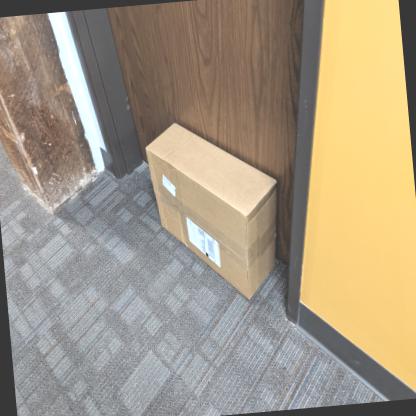

Supplement: Supplemental Information 1 [file peerj-cs-09-1451-s001.zip › train/PXL_20201105_173148569_jpg.rf.b1a0f50296e39ac0ef969220cac58f64.jpg]

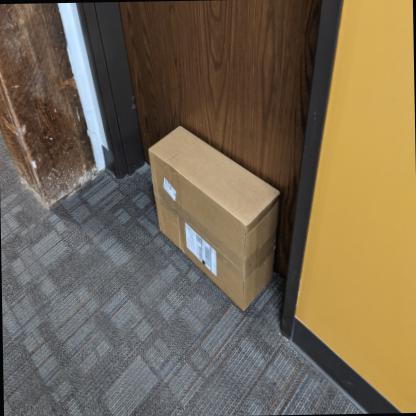

Supplement: Supplemental Information 1 [file peerj-cs-09-1451-s001.zip › train/PXL_20201105_173148569_jpg.rf.bd0e5919a322d8262e471515a0df62a9.jpg]

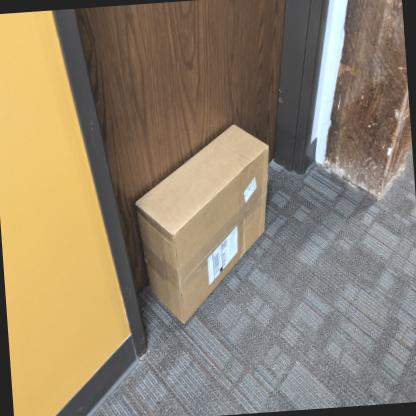

Supplement: Supplemental Information 1 [file peerj-cs-09-1451-s001.zip › train/PXL_20201105_173148569_jpg.rf.dfea357bf308b00b3241a52324e8f836.jpg]

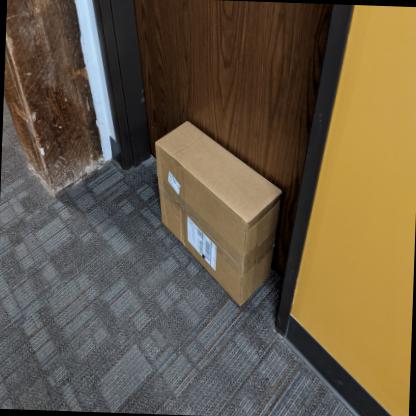

Supplement: Supplemental Information 1 [file peerj-cs-09-1451-s001.zip › train/PXL_20201105_173148569_jpg.rf.fe3f2e53f3402e5ed81bbdcd736d31cf.jpg]

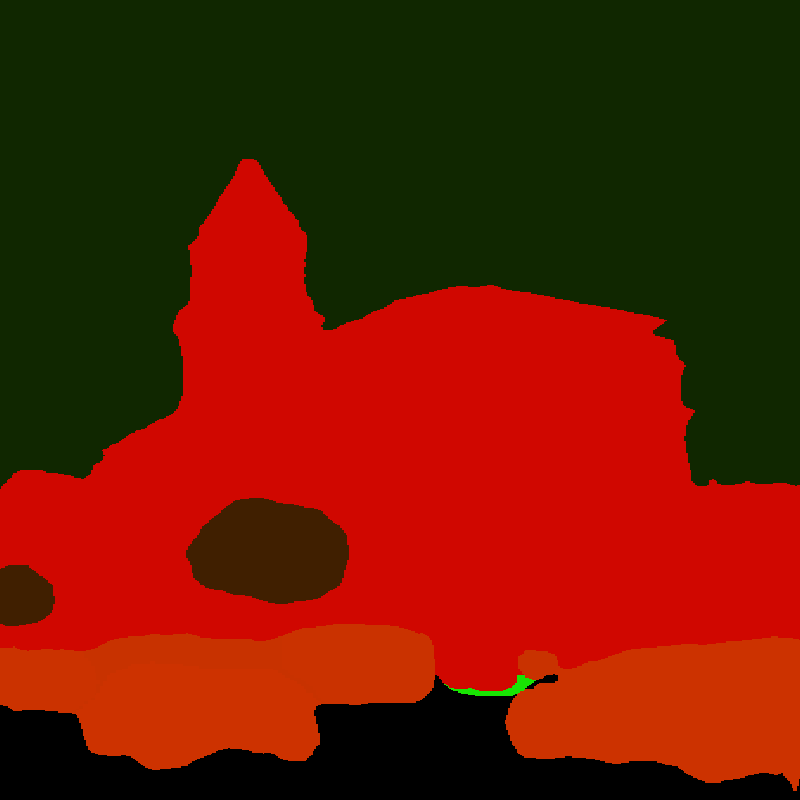

Supplement: Supplemental Information 2 [file peerj-cs-09-1451-s002.zip › ┤·┬δ/data/testdata/dummy_gt_for_vps.png]

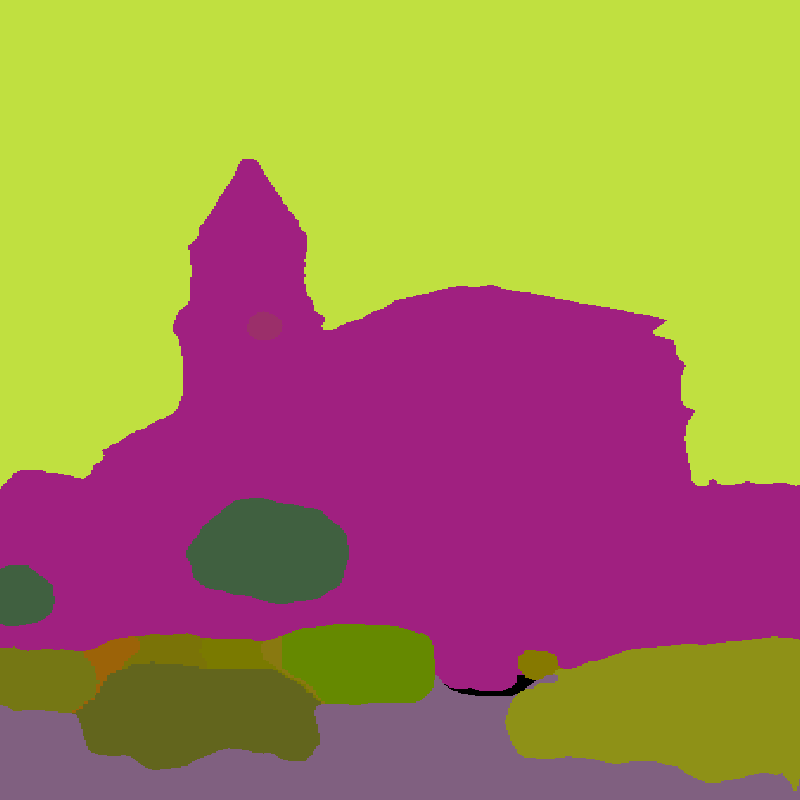

Supplement: Supplemental Information 2 [file peerj-cs-09-1451-s002.zip › ┤·┬δ/data/testdata/dummy_prediction.png]

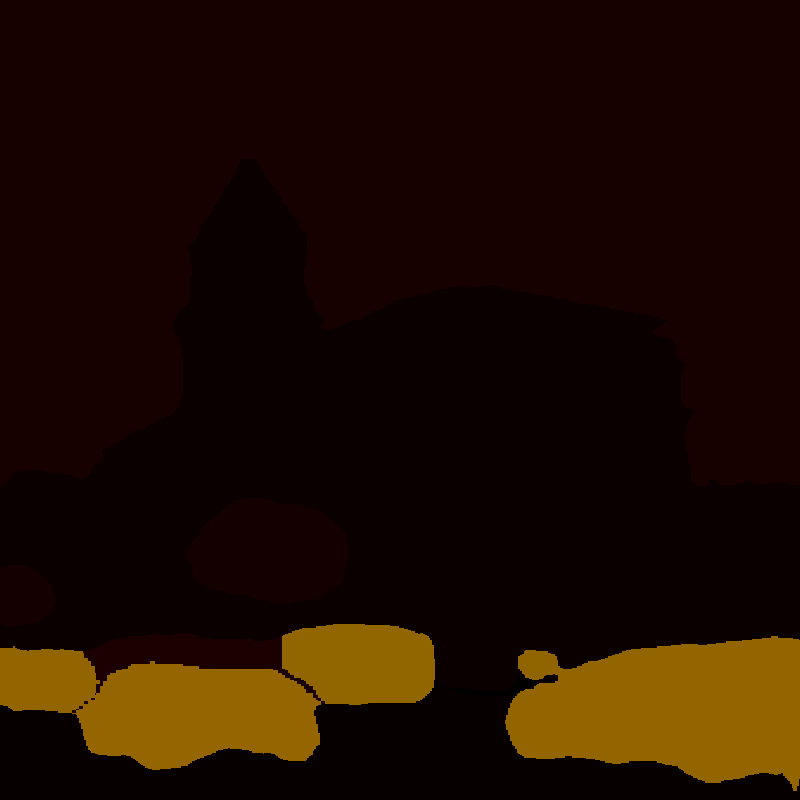

Supplement: Supplemental Information 2 [file peerj-cs-09-1451-s002.zip › ┤·┬δ/data/testdata/gtFine/cityscapes_panoptic_dummy_trainId/dummy_000000_000000_gtFine_panoptic.png]
