# Supplementary figures and images for: Packaging style design based on visual semantic segmentation technology and intelligent cyber physical system (part 1 of 3)
Source: PeerJ Comput Sci. 2023 Jul 10;9:e1451. doi: 10.7717/peerj-cs.1451 (PMC10403159; doi:10.7717/peerj-cs.1451)

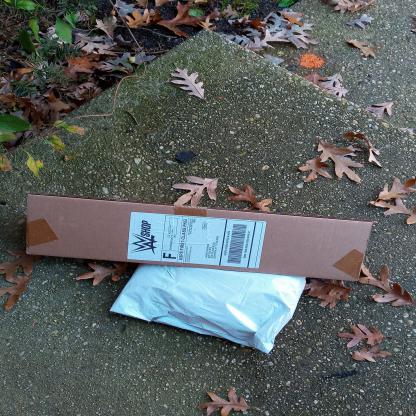

Supplement: Supplemental Information 1 [file peerj-cs-09-1451-s001.zip › test/18_jpg.rf.844374cc4cd6891417b131b55b40f3c5.jpg]

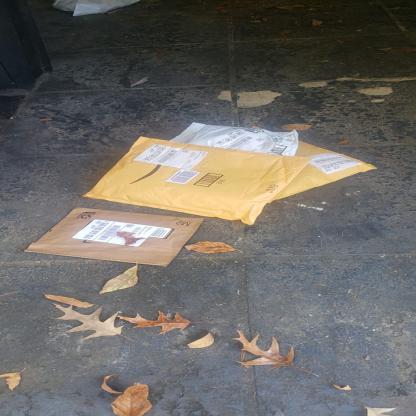

Supplement: Supplemental Information 1 [file peerj-cs-09-1451-s001.zip › test/24_jpg.rf.3b65330eeed9ca9e44ad71cd70f4e52a.jpg]

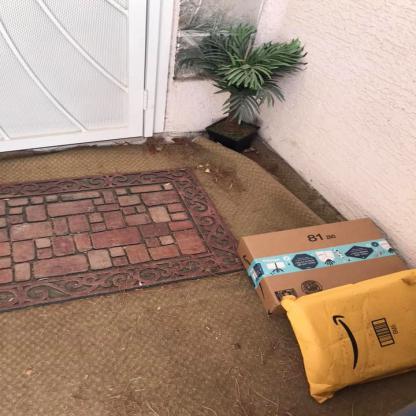

Supplement: Supplemental Information 1 [file peerj-cs-09-1451-s001.zip › test/26_jpg.rf.6aff383da39c46b4a65299ff73681f91.jpg]

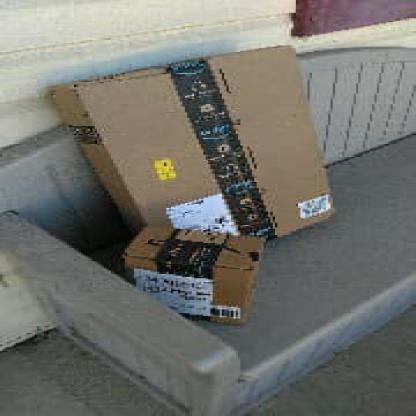

Supplement: Supplemental Information 1 [file peerj-cs-09-1451-s001.zip › test/2_jpg.rf.34f196ef04b288c50b2b066e0cdf3dc8.jpg]

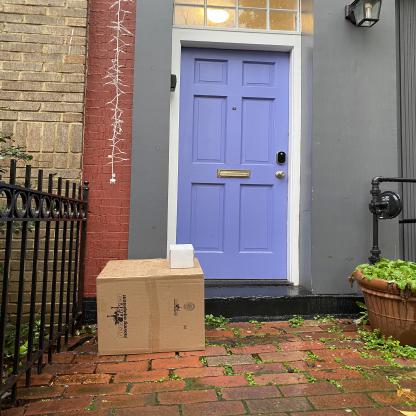

Supplement: Supplemental Information 1 [file peerj-cs-09-1451-s001.zip › test/IMG_6820_jpg.rf.f18338f170168084a6c1cd05c4274f21.jpg]

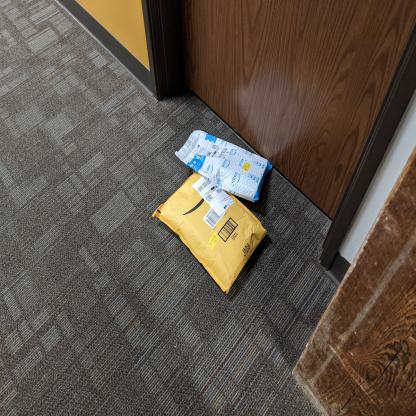

Supplement: Supplemental Information 1 [file peerj-cs-09-1451-s001.zip › test/PXL_20201103_181916758_jpg.rf.b52dd0fe46713fb8ad00cb1aff1140fb.jpg]

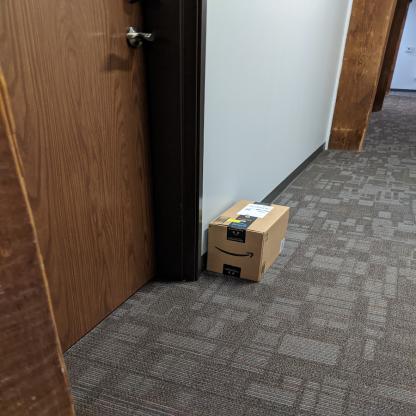

Supplement: Supplemental Information 1 [file peerj-cs-09-1451-s001.zip › test/PXL_20201107_191145720_jpg.rf.a952948ab5ac9a5eac77099ae01cb669.jpg]

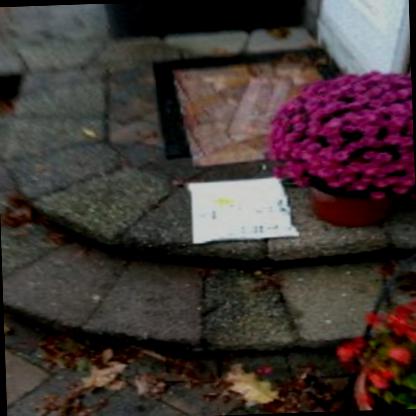

Supplement: Supplemental Information 1 [file peerj-cs-09-1451-s001.zip › train/10_jpg.rf.478e5215e83dd02d3dafb064d8d76f81.jpg]

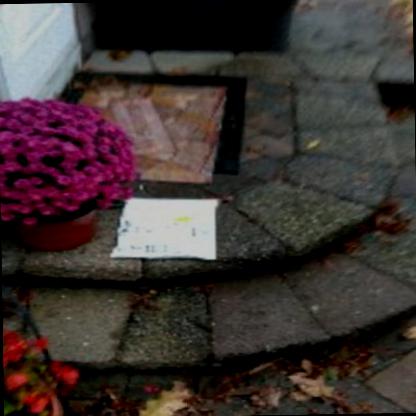

Supplement: Supplemental Information 1 [file peerj-cs-09-1451-s001.zip › train/10_jpg.rf.54d74264a9b638606b4040c92da1b56e.jpg]

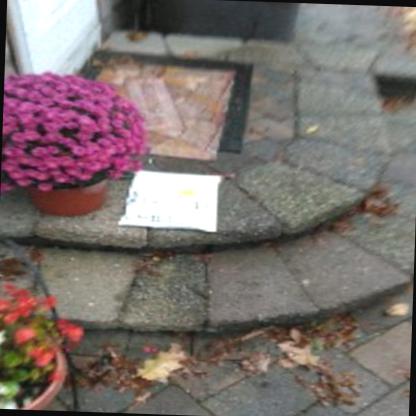

Supplement: Supplemental Information 1 [file peerj-cs-09-1451-s001.zip › train/10_jpg.rf.76d3330c8a94538865d1bcc83fa508d7.jpg]

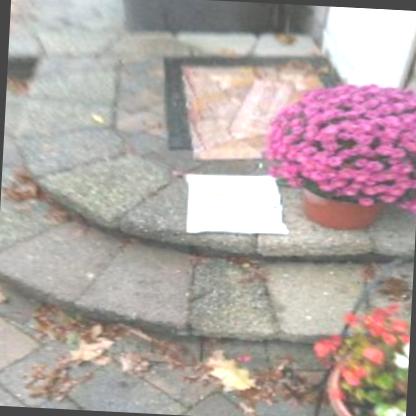

Supplement: Supplemental Information 1 [file peerj-cs-09-1451-s001.zip › train/10_jpg.rf.955ba3cc6477a49811c902329ed30d04.jpg]

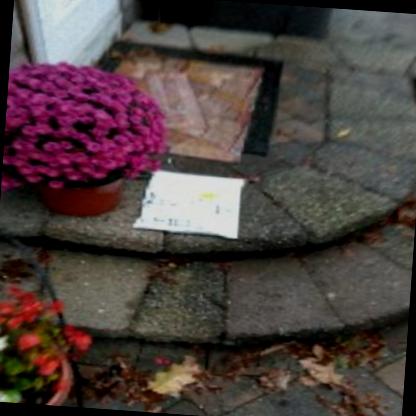

Supplement: Supplemental Information 1 [file peerj-cs-09-1451-s001.zip › train/10_jpg.rf.b4ca45441b633d4acfa785fdbb0ce5f4.jpg]

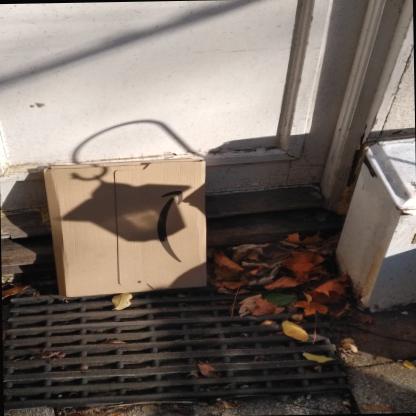

Supplement: Supplemental Information 1 [file peerj-cs-09-1451-s001.zip › train/11_jpg.rf.38225b90f606a87a1fe2a11620fc3809.jpg]

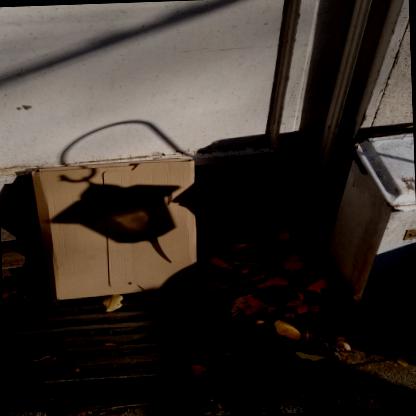

Supplement: Supplemental Information 1 [file peerj-cs-09-1451-s001.zip › train/11_jpg.rf.38b618eb0b13cfa3de3dc112889c9baf.jpg]

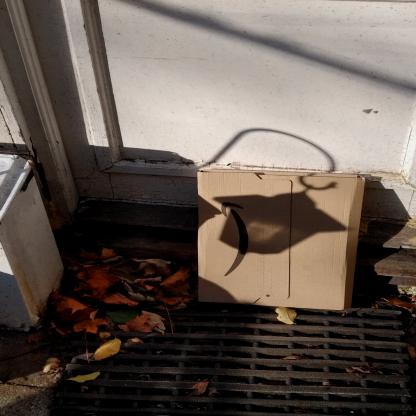

Supplement: Supplemental Information 1 [file peerj-cs-09-1451-s001.zip › train/11_jpg.rf.4e210f34bca47650343668a360f2735d.jpg]

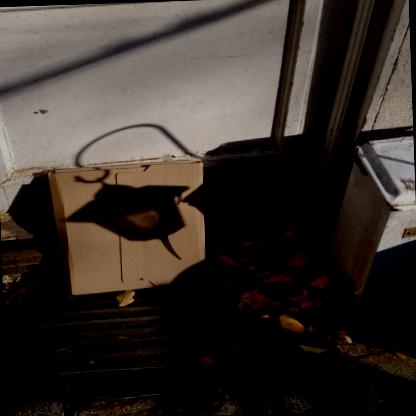

Supplement: Supplemental Information 1 [file peerj-cs-09-1451-s001.zip › train/11_jpg.rf.b0ed27528866c08c400b8ce4137c88c6.jpg]

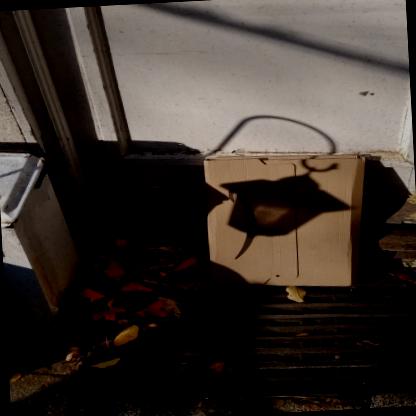

Supplement: Supplemental Information 1 [file peerj-cs-09-1451-s001.zip › train/11_jpg.rf.dd8514645c93a5159c45258ea336bc2e.jpg]

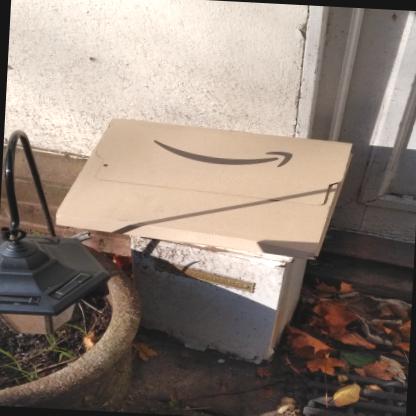

Supplement: Supplemental Information 1 [file peerj-cs-09-1451-s001.zip › train/12_jpg.rf.0c3dd93746ad7a2950f152998e2cd162.jpg]

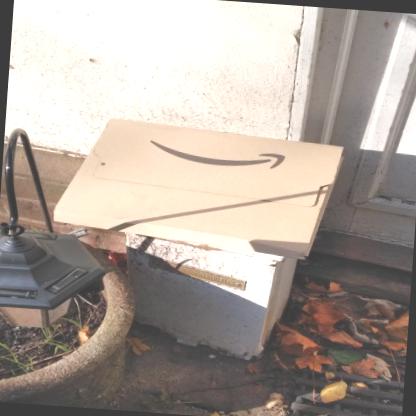

Supplement: Supplemental Information 1 [file peerj-cs-09-1451-s001.zip › train/12_jpg.rf.1a534dbc51f6ad7fcbde9bde9061e951.jpg]

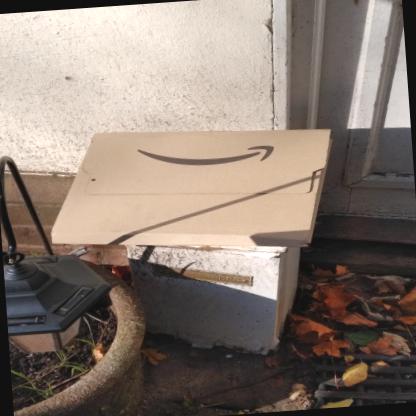

Supplement: Supplemental Information 1 [file peerj-cs-09-1451-s001.zip › train/12_jpg.rf.6ab34c0f46751cbbd4308165da10c0e9.jpg]

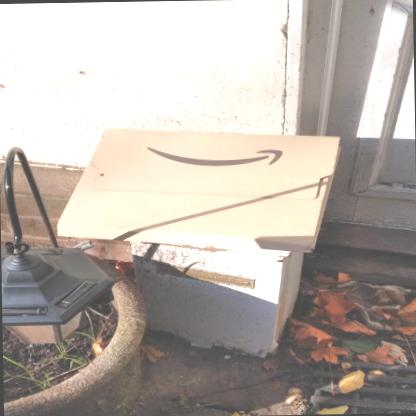

Supplement: Supplemental Information 1 [file peerj-cs-09-1451-s001.zip › train/12_jpg.rf.f472e4ef65a6ba54096d613aefe9d040.jpg]

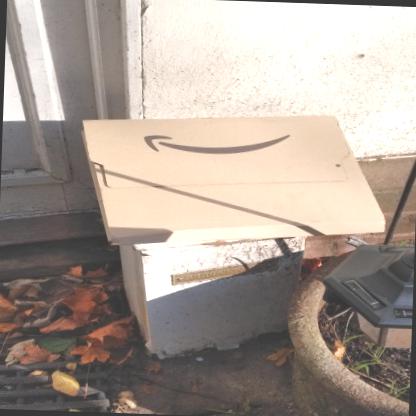

Supplement: Supplemental Information 1 [file peerj-cs-09-1451-s001.zip › train/12_jpg.rf.fb71b320057654212e74591f6f87fbd3.jpg]

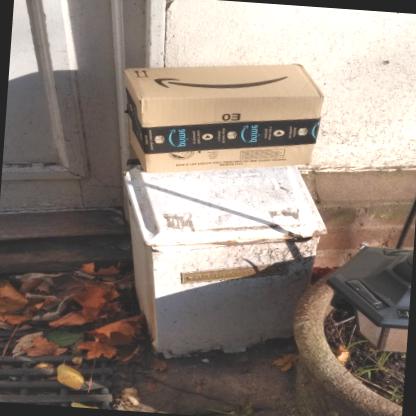

Supplement: Supplemental Information 1 [file peerj-cs-09-1451-s001.zip › train/13_jpg.rf.1df0097dfbadf62952f1879702b4dc1f.jpg]

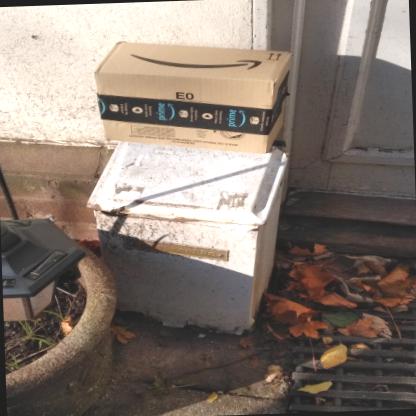

Supplement: Supplemental Information 1 [file peerj-cs-09-1451-s001.zip › train/13_jpg.rf.5d45bd56b897aebaf3ac292cf78ebed1.jpg]

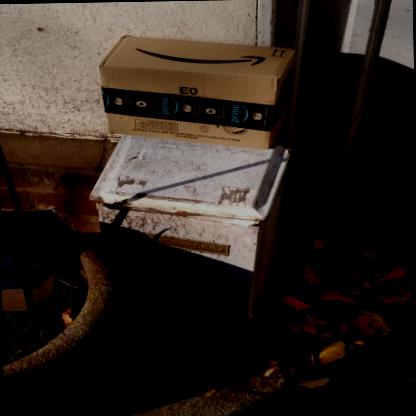

Supplement: Supplemental Information 1 [file peerj-cs-09-1451-s001.zip › train/13_jpg.rf.90fd6952968e6460ef1c0f96ade0bfe8.jpg]

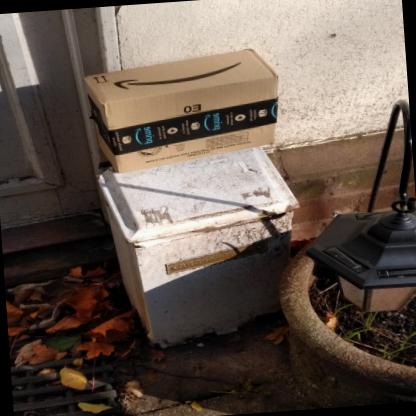

Supplement: Supplemental Information 1 [file peerj-cs-09-1451-s001.zip › train/13_jpg.rf.9a3e86b0a72706a3007e4cd26c5059d6.jpg]

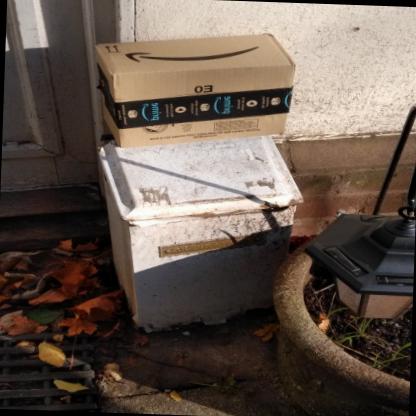

Supplement: Supplemental Information 1 [file peerj-cs-09-1451-s001.zip › train/13_jpg.rf.a9f6d6e9d93b9fd641e11225a7893229.jpg]

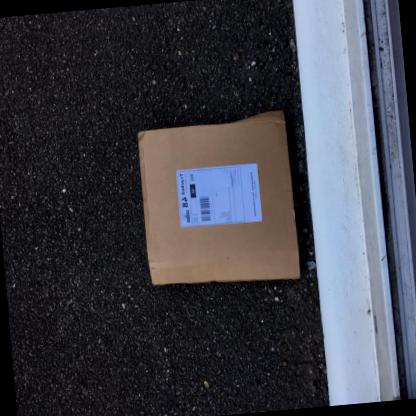

Supplement: Supplemental Information 1 [file peerj-cs-09-1451-s001.zip › train/14_jpg.rf.468465b31cdf08e138e8a774f9c72b73.jpg]

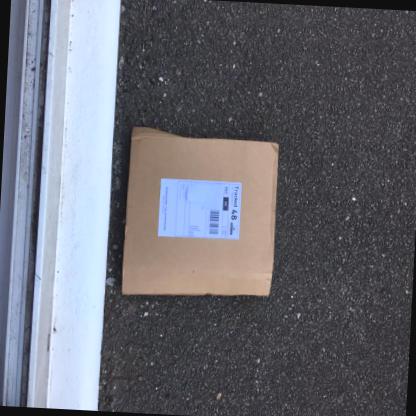

Supplement: Supplemental Information 1 [file peerj-cs-09-1451-s001.zip › train/14_jpg.rf.5a5f07927fb4d0eabe68c594654d0961.jpg]

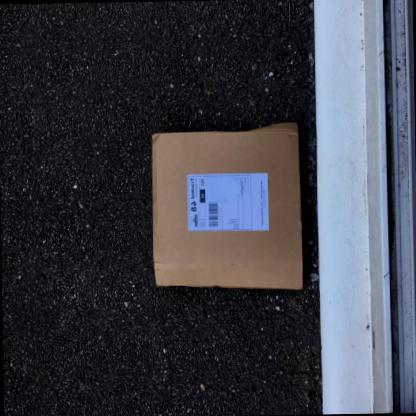

Supplement: Supplemental Information 1 [file peerj-cs-09-1451-s001.zip › train/14_jpg.rf.652042a0d26ba51feada836024b1f935.jpg]

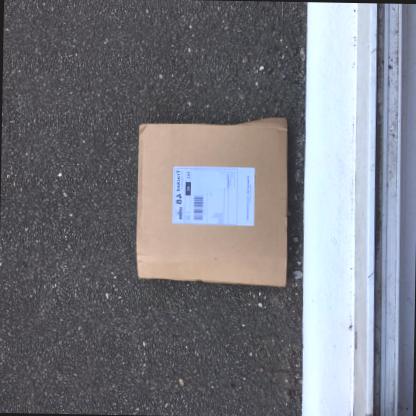

Supplement: Supplemental Information 1 [file peerj-cs-09-1451-s001.zip › train/14_jpg.rf.c034732d584163f89a993e4ed05382a5.jpg]

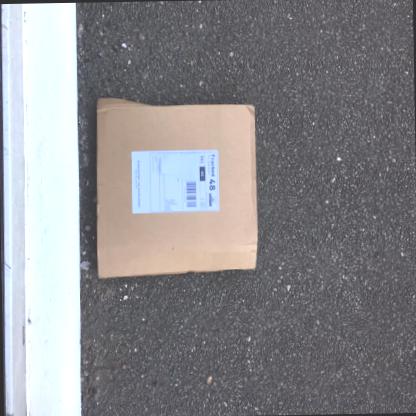

Supplement: Supplemental Information 1 [file peerj-cs-09-1451-s001.zip › train/14_jpg.rf.cac542444734dbed55c647a71ef20afd.jpg]

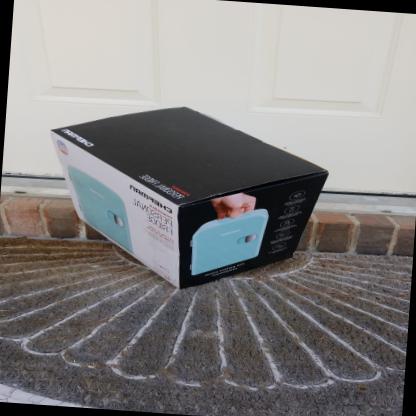

Supplement: Supplemental Information 1 [file peerj-cs-09-1451-s001.zip › train/15_jpg.rf.110eabea1cd148b9f5b2a97e0cfda81a.jpg]

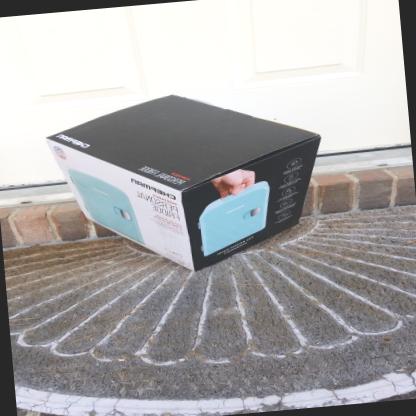

Supplement: Supplemental Information 1 [file peerj-cs-09-1451-s001.zip › train/15_jpg.rf.7b29646ddf3f4b7b3da74fd311fa997d.jpg]

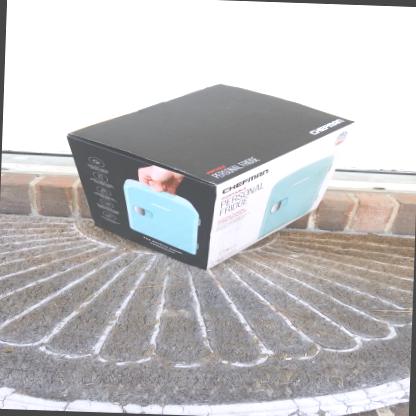

Supplement: Supplemental Information 1 [file peerj-cs-09-1451-s001.zip › train/15_jpg.rf.7e63c7cff39c295d57563989f85c6480.jpg]

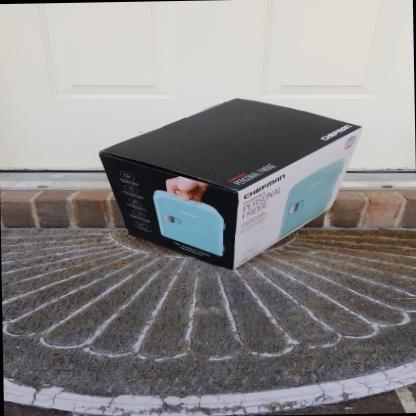

Supplement: Supplemental Information 1 [file peerj-cs-09-1451-s001.zip › train/15_jpg.rf.ac55b19d04f3a3b0a371babb037d0ffb.jpg]

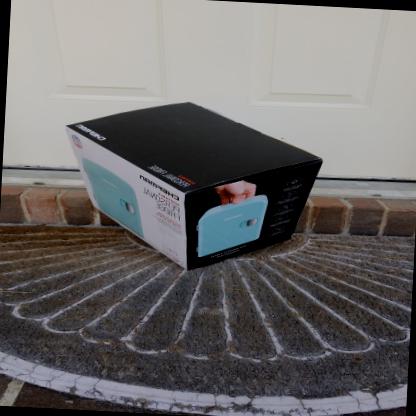

Supplement: Supplemental Information 1 [file peerj-cs-09-1451-s001.zip › train/15_jpg.rf.b2237acc691aa6d9a5e3bd61aa1c1843.jpg]

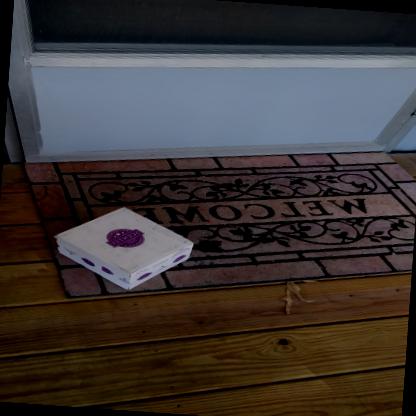

Supplement: Supplemental Information 1 [file peerj-cs-09-1451-s001.zip › train/16_jpg.rf.0b9d96c0d3045e34c764e8b661256964.jpg]

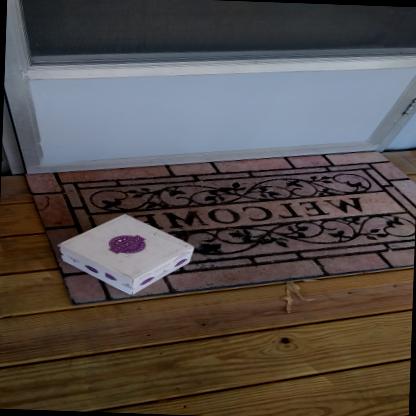

Supplement: Supplemental Information 1 [file peerj-cs-09-1451-s001.zip › train/16_jpg.rf.2f8eeab7e26ccd927d838d9c9cfe5000.jpg]

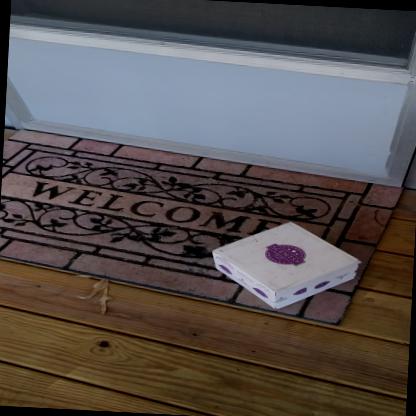

Supplement: Supplemental Information 1 [file peerj-cs-09-1451-s001.zip › train/16_jpg.rf.3fb3d14906230b5c58783db0c8ca2d56.jpg]

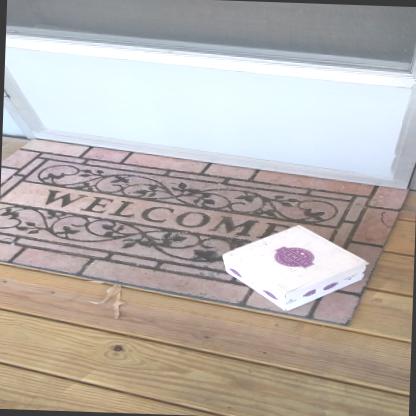

Supplement: Supplemental Information 1 [file peerj-cs-09-1451-s001.zip › train/16_jpg.rf.591a5314f007fdf2c5cbb266a6c62ae7.jpg]

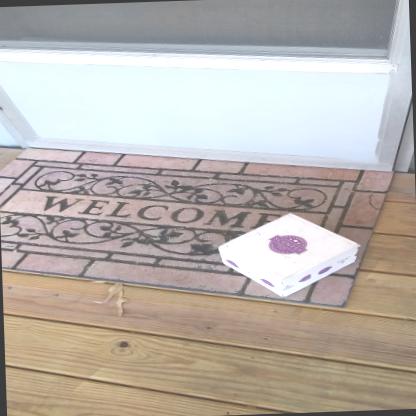

Supplement: Supplemental Information 1 [file peerj-cs-09-1451-s001.zip › train/16_jpg.rf.716006d8932990f728226303e7d4aa40.jpg]

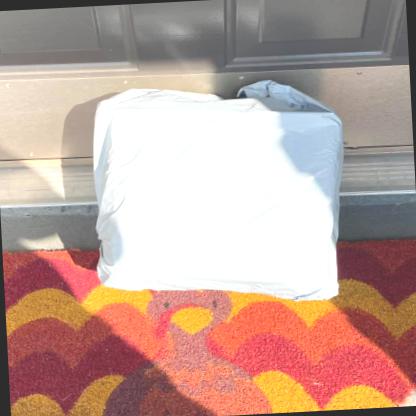

Supplement: Supplemental Information 1 [file peerj-cs-09-1451-s001.zip › train/19_jpg.rf.11286df800be3625622390657d41a64e.jpg]

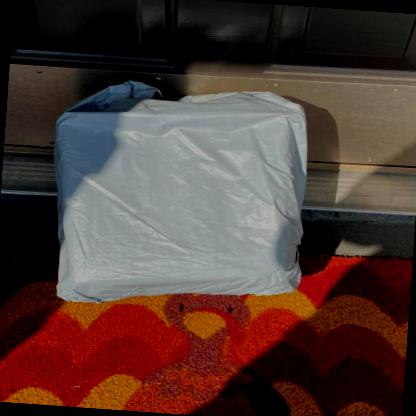

Supplement: Supplemental Information 1 [file peerj-cs-09-1451-s001.zip › train/19_jpg.rf.233920e03e111b3fe943161909c58aa0.jpg]

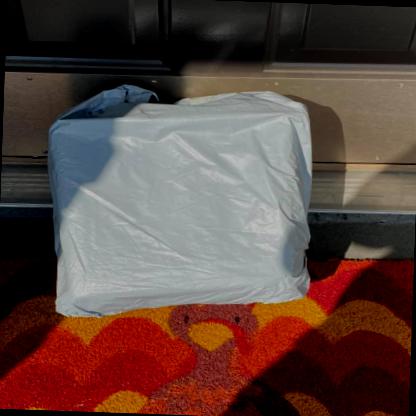

Supplement: Supplemental Information 1 [file peerj-cs-09-1451-s001.zip › train/19_jpg.rf.be8668ef2a68b539159fd7ddd2452b08.jpg]

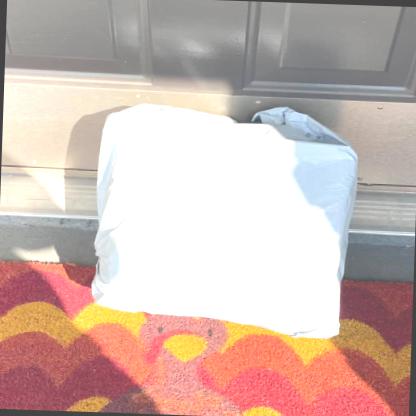

Supplement: Supplemental Information 1 [file peerj-cs-09-1451-s001.zip › train/19_jpg.rf.c35c8d1570db5b45b439c4b60bb357ab.jpg]

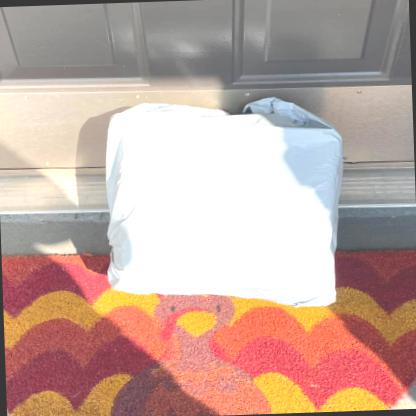

Supplement: Supplemental Information 1 [file peerj-cs-09-1451-s001.zip › train/19_jpg.rf.d6839e4fe618d41b12ff637ad0dc8aec.jpg]

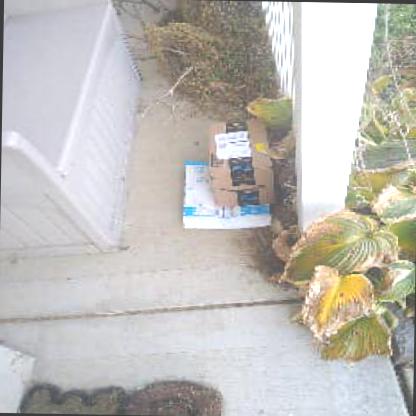

Supplement: Supplemental Information 1 [file peerj-cs-09-1451-s001.zip › train/1_jpg.rf.2aac586533ba280a3f82d6d223aa4149.jpg]

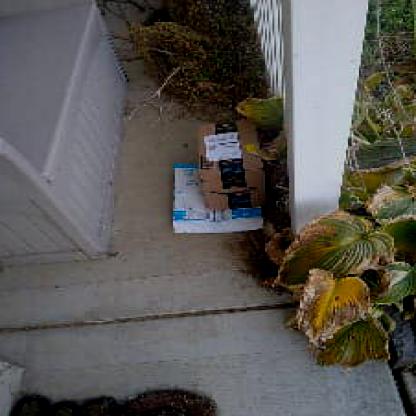

Supplement: Supplemental Information 1 [file peerj-cs-09-1451-s001.zip › train/1_jpg.rf.34a22137a2d7565bd66bd6b4767aacc5.jpg]

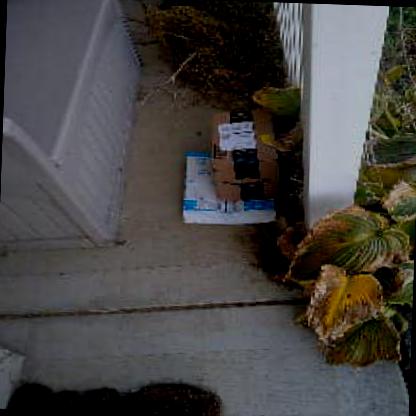

Supplement: Supplemental Information 1 [file peerj-cs-09-1451-s001.zip › train/1_jpg.rf.371fa365dbe538aa610c983bc0beb9ce.jpg]

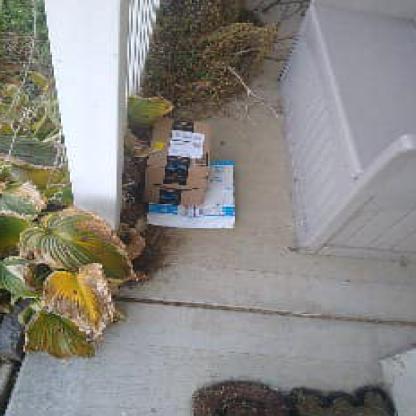

Supplement: Supplemental Information 1 [file peerj-cs-09-1451-s001.zip › train/1_jpg.rf.6087e08ee049d129519aacf4517918b5.jpg]

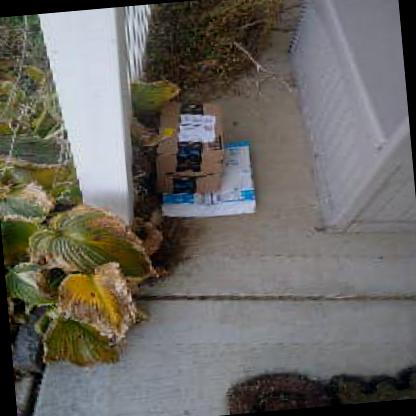

Supplement: Supplemental Information 1 [file peerj-cs-09-1451-s001.zip › train/1_jpg.rf.8975d4477eea9eace54e807b36857765.jpg]

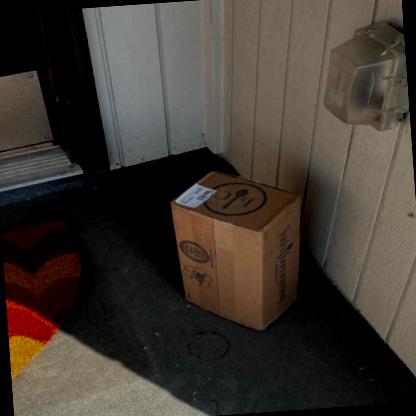

Supplement: Supplemental Information 1 [file peerj-cs-09-1451-s001.zip › train/20_jpg.rf.01797f329ab6636c644ccfde8db679e2.jpg]

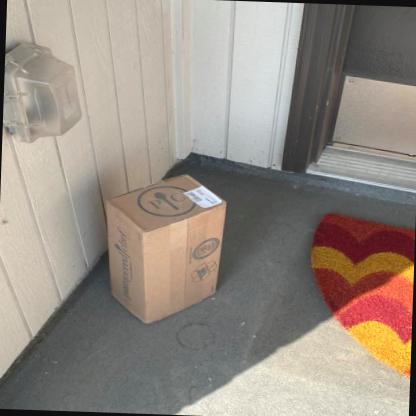

Supplement: Supplemental Information 1 [file peerj-cs-09-1451-s001.zip › train/20_jpg.rf.0c599cec4351372ffe6da1bfde8fd104.jpg]

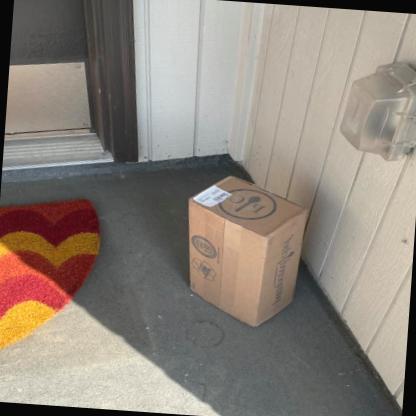

Supplement: Supplemental Information 1 [file peerj-cs-09-1451-s001.zip › train/20_jpg.rf.22897fa6c319b800de4807ddd704f789.jpg]

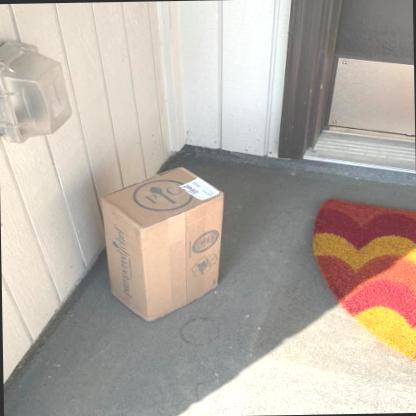

Supplement: Supplemental Information 1 [file peerj-cs-09-1451-s001.zip › train/20_jpg.rf.6b5228b18ff1e0a7a07bc540363bba97.jpg]

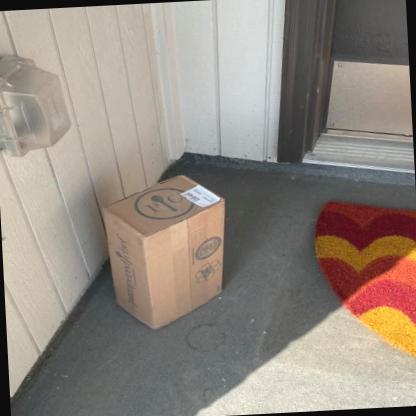

Supplement: Supplemental Information 1 [file peerj-cs-09-1451-s001.zip › train/20_jpg.rf.b33621cfaa4a7c61c387d7d741907b38.jpg]

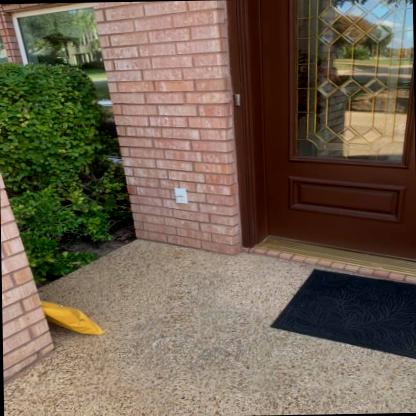

Supplement: Supplemental Information 1 [file peerj-cs-09-1451-s001.zip › train/21_jpg.rf.0a3f4dffd99d3023f2defb86d459ad62.jpg]

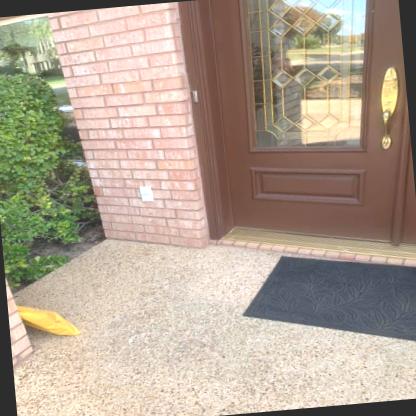

Supplement: Supplemental Information 1 [file peerj-cs-09-1451-s001.zip › train/21_jpg.rf.39c52a07b68d0b43ebd06f37f839a57d.jpg]

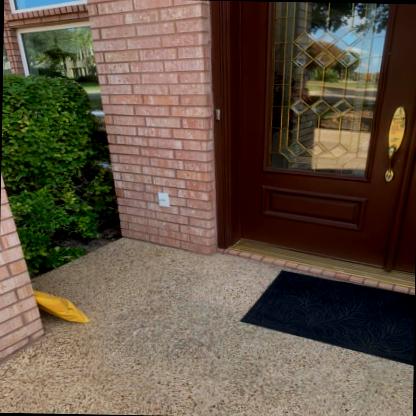

Supplement: Supplemental Information 1 [file peerj-cs-09-1451-s001.zip › train/21_jpg.rf.a5af34f235290801f42e947af81f5c0c.jpg]

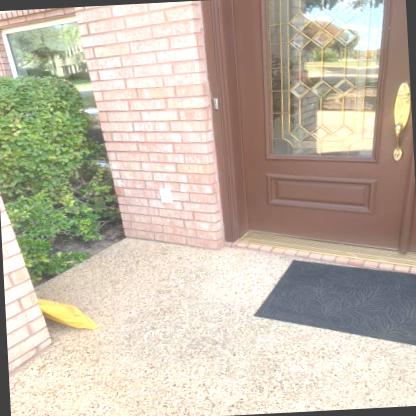

Supplement: Supplemental Information 1 [file peerj-cs-09-1451-s001.zip › train/21_jpg.rf.c1fbf13e2126054333201560e60b6e68.jpg]

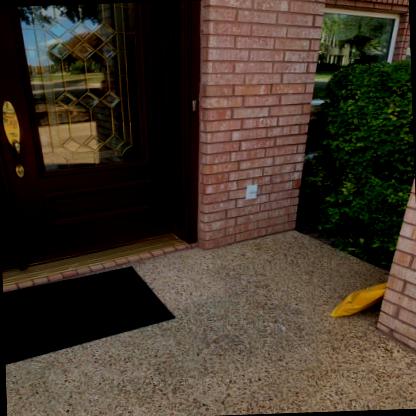

Supplement: Supplemental Information 1 [file peerj-cs-09-1451-s001.zip › train/21_jpg.rf.dc0ae9059065984bf8edaeb47939e4a2.jpg]

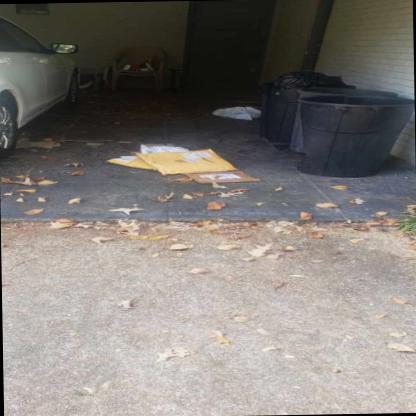

Supplement: Supplemental Information 1 [file peerj-cs-09-1451-s001.zip › train/23_jpg.rf.14bd49e759598129cfd651fbd56a450c.jpg]

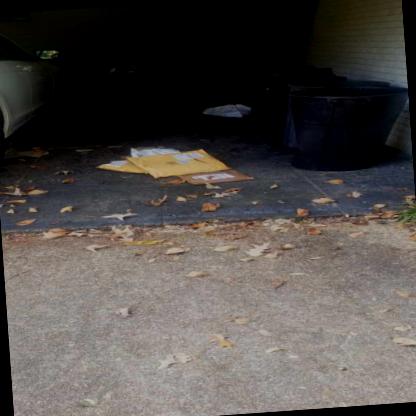

Supplement: Supplemental Information 1 [file peerj-cs-09-1451-s001.zip › train/23_jpg.rf.2baa76a1992a597eadebe4c05f479374.jpg]

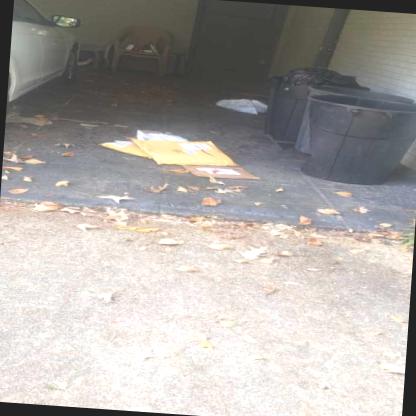

Supplement: Supplemental Information 1 [file peerj-cs-09-1451-s001.zip › train/23_jpg.rf.4dad4e0808e25edaa22fdd766bc377f6.jpg]

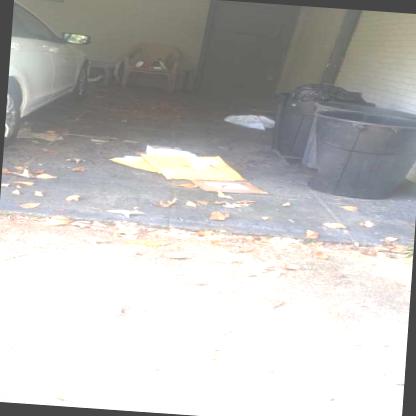

Supplement: Supplemental Information 1 [file peerj-cs-09-1451-s001.zip › train/23_jpg.rf.5b04e628e71de0a57e09c4b66aeb54e5.jpg]

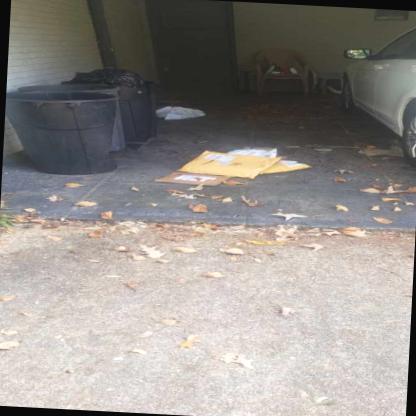

Supplement: Supplemental Information 1 [file peerj-cs-09-1451-s001.zip › train/23_jpg.rf.e00a0dc4e124fb7810b93686c0fe5cdc.jpg]

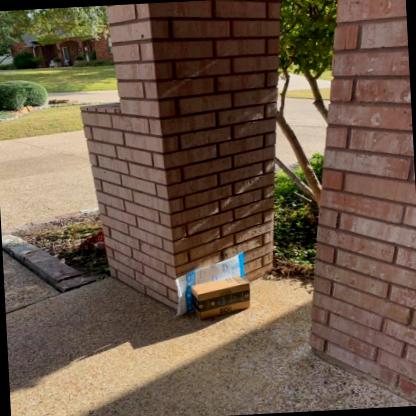

Supplement: Supplemental Information 1 [file peerj-cs-09-1451-s001.zip › train/25_jpg.rf.136545cbe252197d0b118656ac657034.jpg]

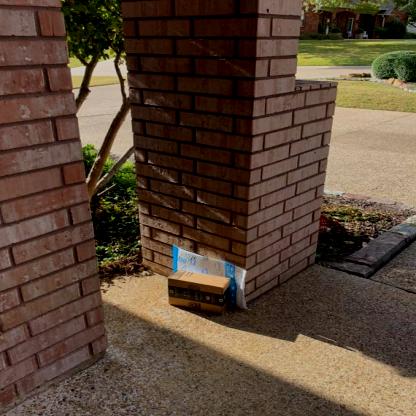

Supplement: Supplemental Information 1 [file peerj-cs-09-1451-s001.zip › train/25_jpg.rf.37867f7fb310ad5bc842e97fa401a0de.jpg]

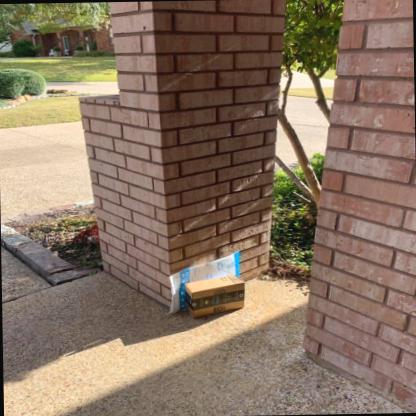

Supplement: Supplemental Information 1 [file peerj-cs-09-1451-s001.zip › train/25_jpg.rf.56faef70c2cfe9642c345ae5f5288126.jpg]

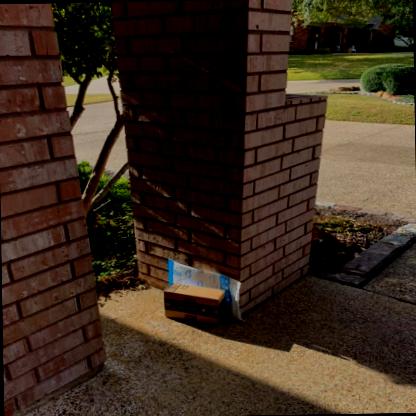

Supplement: Supplemental Information 1 [file peerj-cs-09-1451-s001.zip › train/25_jpg.rf.6d56a918545d311435754cd5a3a33c7d.jpg]

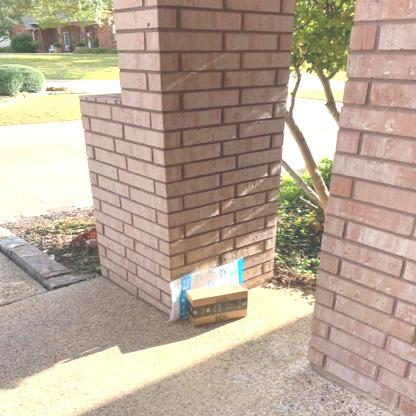

Supplement: Supplemental Information 1 [file peerj-cs-09-1451-s001.zip › train/25_jpg.rf.d238a7cec35f99876a7313b53074ce67.jpg]

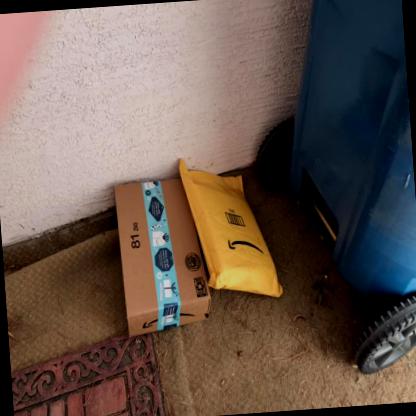

Supplement: Supplemental Information 1 [file peerj-cs-09-1451-s001.zip › train/27_jpg.rf.294fdc90f7f03af033f520e21fd78108.jpg]

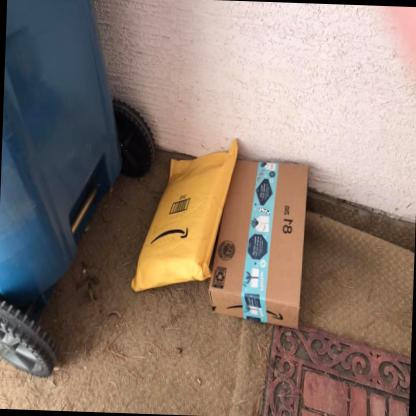

Supplement: Supplemental Information 1 [file peerj-cs-09-1451-s001.zip › train/27_jpg.rf.2dc976b255102ad647edb16aa106972e.jpg]

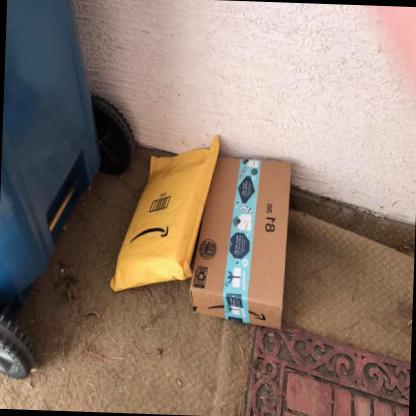

Supplement: Supplemental Information 1 [file peerj-cs-09-1451-s001.zip › train/27_jpg.rf.460c085da0b780ccaabe8fa1ab7cd470.jpg]

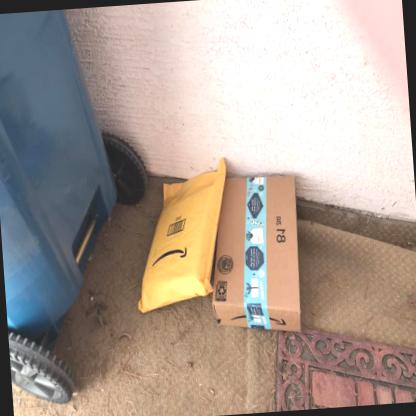

Supplement: Supplemental Information 1 [file peerj-cs-09-1451-s001.zip › train/27_jpg.rf.494d52f7b9a3beb7af29e0e58d1542bd.jpg]

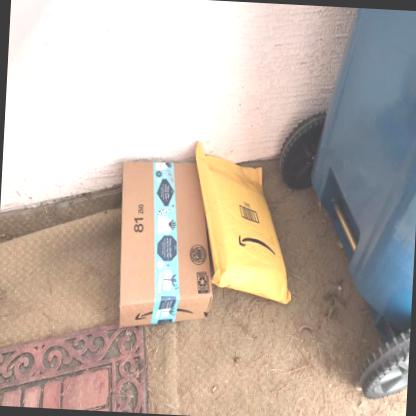

Supplement: Supplemental Information 1 [file peerj-cs-09-1451-s001.zip › train/27_jpg.rf.ec06d5ebe2b85f7306df1d8ba8ccf1b4.jpg]

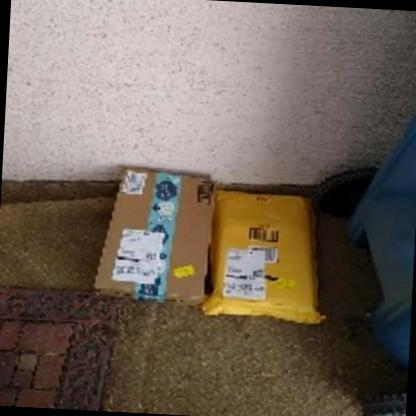

Supplement: Supplemental Information 1 [file peerj-cs-09-1451-s001.zip › train/28_jpg.rf.1fd2549e819ea3089b841e1ca4af3da9.jpg]

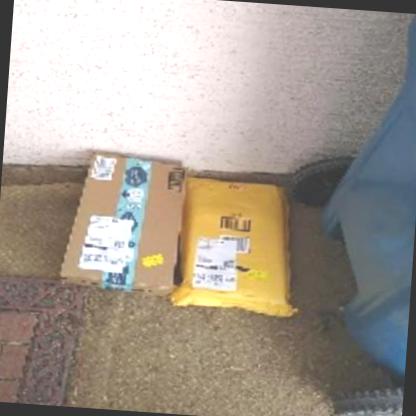

Supplement: Supplemental Information 1 [file peerj-cs-09-1451-s001.zip › train/28_jpg.rf.3bd4c1b3e53a5d5d22a9fca6f21207c5.jpg]

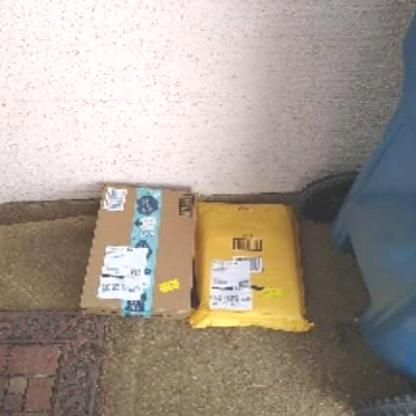

Supplement: Supplemental Information 1 [file peerj-cs-09-1451-s001.zip › train/28_jpg.rf.80e51e099cf80e896508c42a5e1c3ee1.jpg]

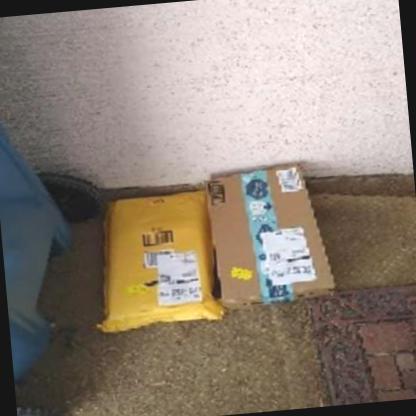

Supplement: Supplemental Information 1 [file peerj-cs-09-1451-s001.zip › train/28_jpg.rf.e288b3e277c80491721c182e87cb4462.jpg]

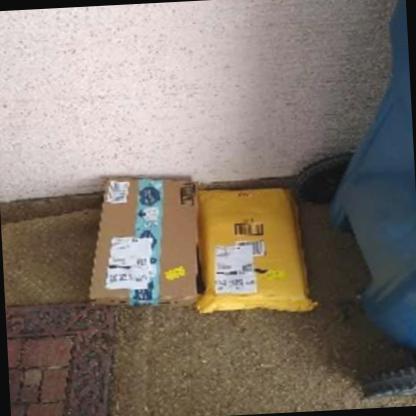

Supplement: Supplemental Information 1 [file peerj-cs-09-1451-s001.zip › train/28_jpg.rf.fef1c8a5c15a9e677b1364a1a60cedf2.jpg]

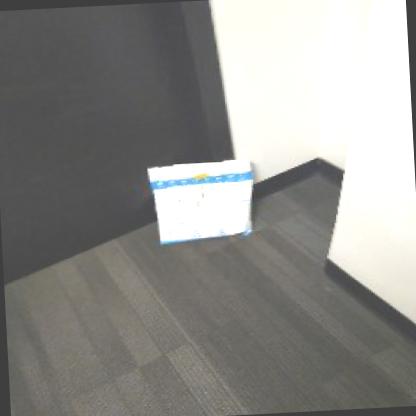

Supplement: Supplemental Information 1 [file peerj-cs-09-1451-s001.zip › train/3_jpg.rf.1678675ccf57faa292efc80289ab30bf.jpg]

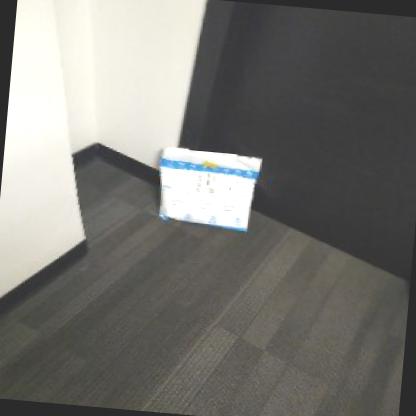

Supplement: Supplemental Information 1 [file peerj-cs-09-1451-s001.zip › train/3_jpg.rf.3246b7696c4c92fb12f900a5f4c3b25a.jpg]

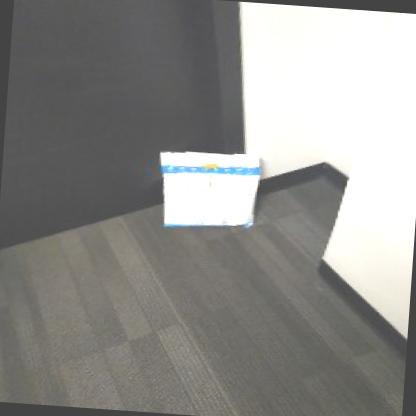

Supplement: Supplemental Information 1 [file peerj-cs-09-1451-s001.zip › train/3_jpg.rf.564896211c403b76b12623182ec440b7.jpg]

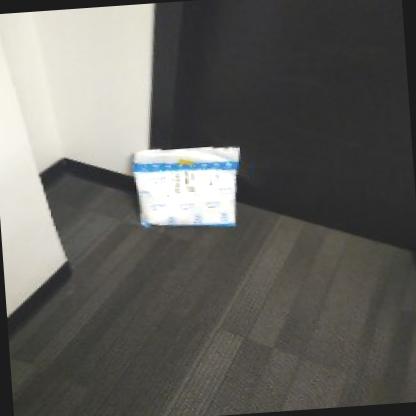

Supplement: Supplemental Information 1 [file peerj-cs-09-1451-s001.zip › train/3_jpg.rf.b540bb62b3e31a4b9ef65fd6f3b09383.jpg]

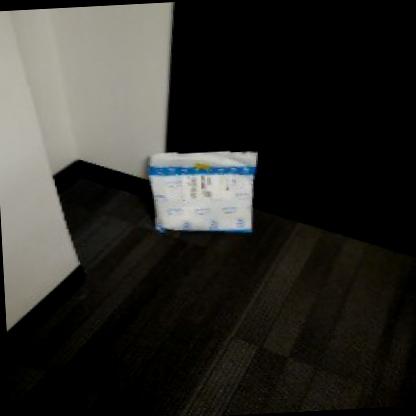

Supplement: Supplemental Information 1 [file peerj-cs-09-1451-s001.zip › train/3_jpg.rf.eb1d9f84db2a88f3d13ae8b6a1d64fd9.jpg]

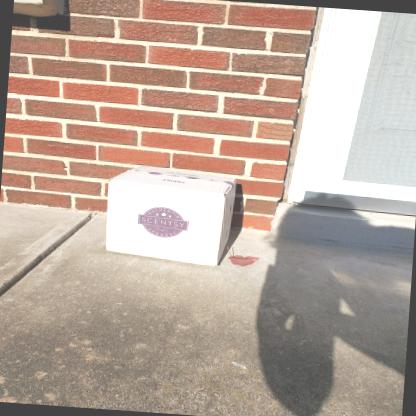

Supplement: Supplemental Information 1 [file peerj-cs-09-1451-s001.zip › train/4_jpg.rf.7ad86b683b150ee3e0cfb69a9e0ee62b.jpg]

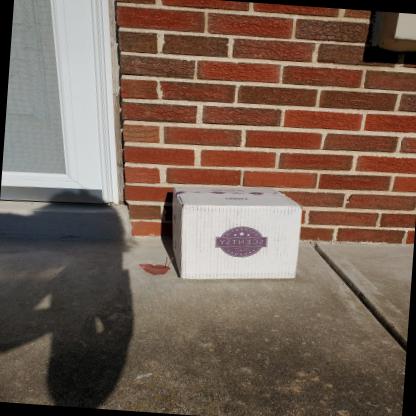

Supplement: Supplemental Information 1 [file peerj-cs-09-1451-s001.zip › train/4_jpg.rf.7e558188ed088c0070aae630b7551fbd.jpg]

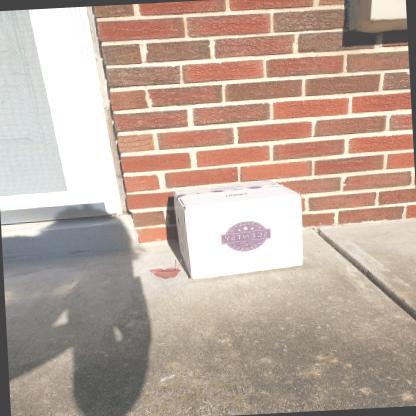

Supplement: Supplemental Information 1 [file peerj-cs-09-1451-s001.zip › train/4_jpg.rf.c4c099c7493368c7fb3805811b02f2f0.jpg]

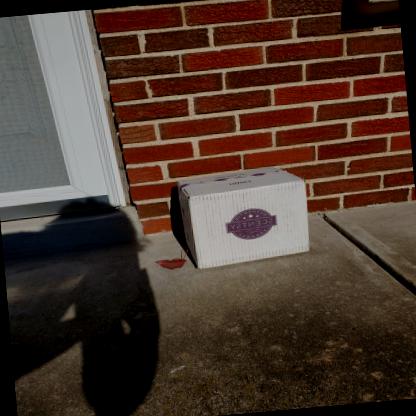

Supplement: Supplemental Information 1 [file peerj-cs-09-1451-s001.zip › train/4_jpg.rf.f1785c9bf792ae76ae3708d214e9f18c.jpg]

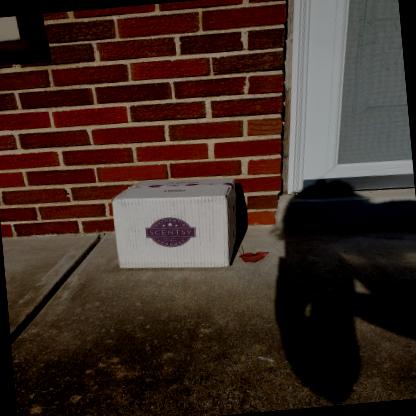

Supplement: Supplemental Information 1 [file peerj-cs-09-1451-s001.zip › train/4_jpg.rf.f36cbbe1194784807ec8e271d92cc120.jpg]

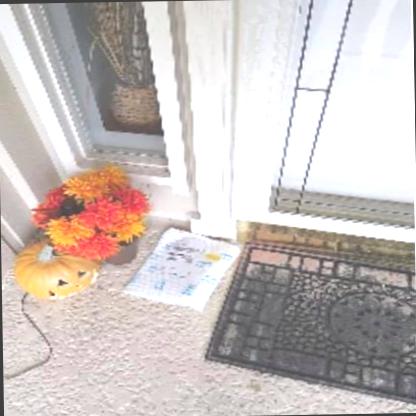

Supplement: Supplemental Information 1 [file peerj-cs-09-1451-s001.zip › train/6_jpg.rf.2309951a5e3ffb379e3bd889523bee7b.jpg]

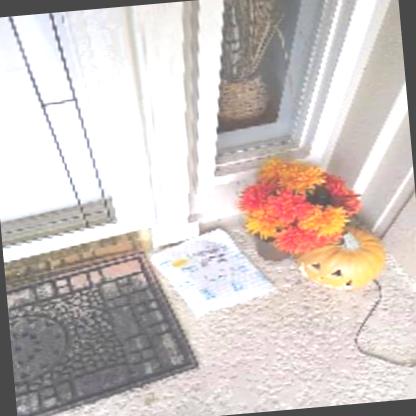

Supplement: Supplemental Information 1 [file peerj-cs-09-1451-s001.zip › train/6_jpg.rf.5cfd2048d681f0c9a12b668d10670577.jpg]

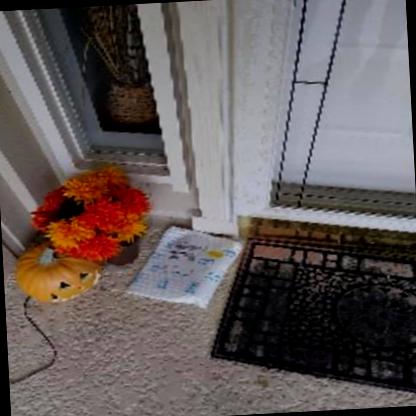

Supplement: Supplemental Information 1 [file peerj-cs-09-1451-s001.zip › train/6_jpg.rf.78aa6bdba45c379d3d082212ad5cd924.jpg]

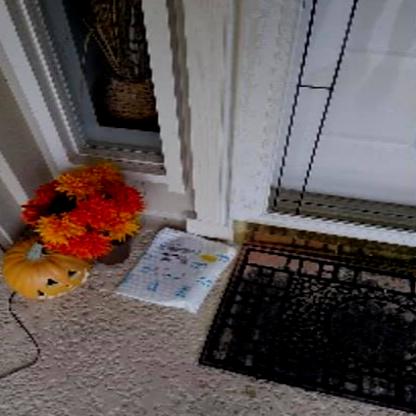

Supplement: Supplemental Information 1 [file peerj-cs-09-1451-s001.zip › train/6_jpg.rf.945167f3e60172a4a7ccb2d2f1747fb2.jpg]

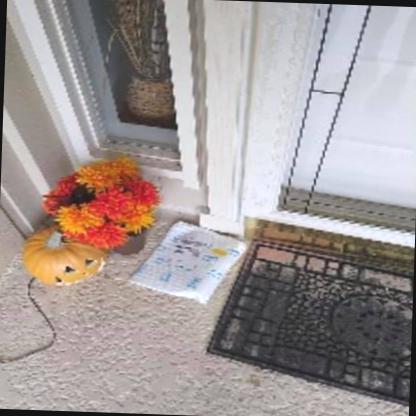

Supplement: Supplemental Information 1 [file peerj-cs-09-1451-s001.zip › train/6_jpg.rf.d803e7fe03f9a532a42a652754d052a2.jpg]

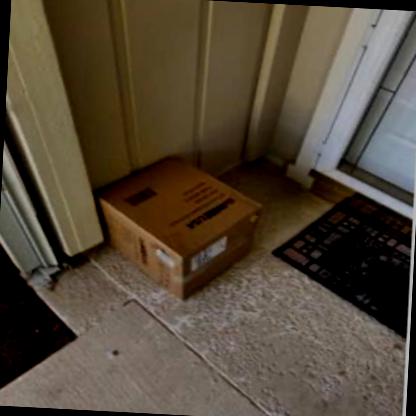

Supplement: Supplemental Information 1 [file peerj-cs-09-1451-s001.zip › train/7_jpg.rf.095ebe6aa1e9e1355f06131be0d1ab93.jpg]

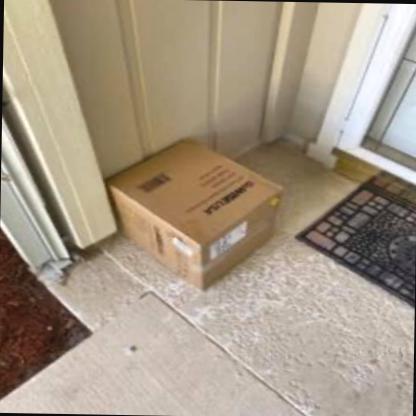

Supplement: Supplemental Information 1 [file peerj-cs-09-1451-s001.zip › train/7_jpg.rf.1629ae54984075c24706153791bd02c2.jpg]

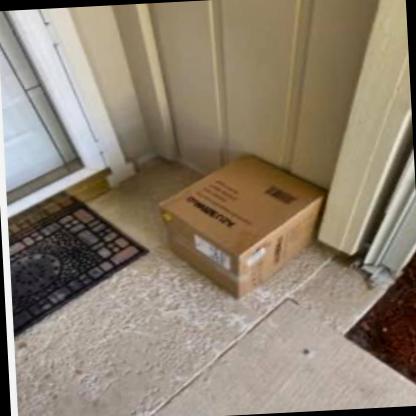

Supplement: Supplemental Information 1 [file peerj-cs-09-1451-s001.zip › train/7_jpg.rf.30825c2771358e24fe81e7708e750c74.jpg]
