# Supplementary figures and images for: Packaging style design based on visual semantic segmentation technology and intelligent cyber physical system (part 3 of 3)
Source: PeerJ Comput Sci. 2023 Jul 10;9:e1451. doi: 10.7717/peerj-cs.1451 (PMC10403159; doi:10.7717/peerj-cs.1451)

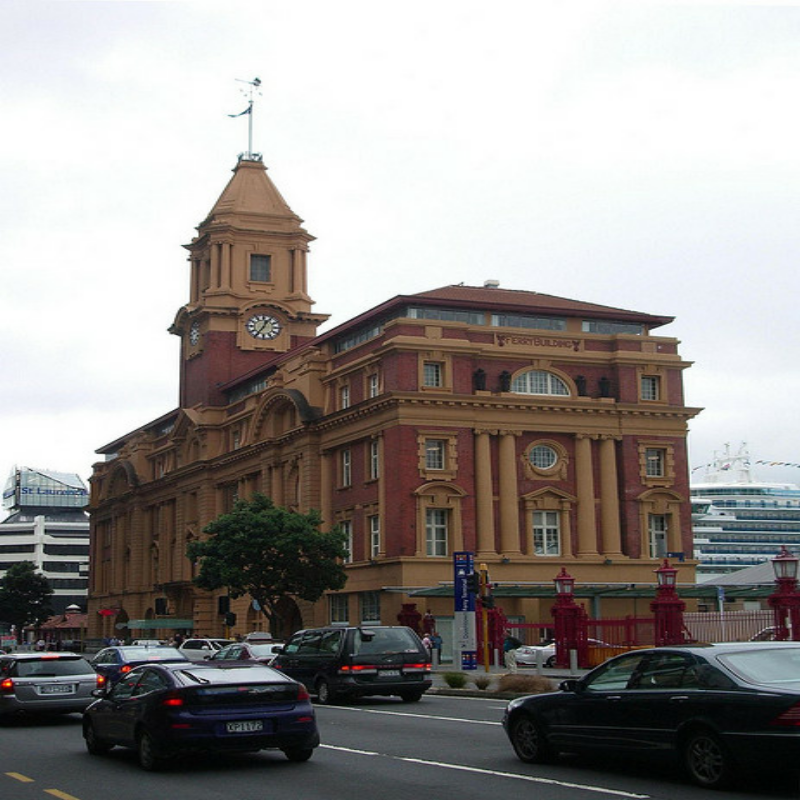

Supplement: Supplemental Information 2 [file peerj-cs-09-1451-s002.zip › ┤·┬δ/data/testdata/leftImg8bit/dummy_000000_000000_leftImg8bit.png]

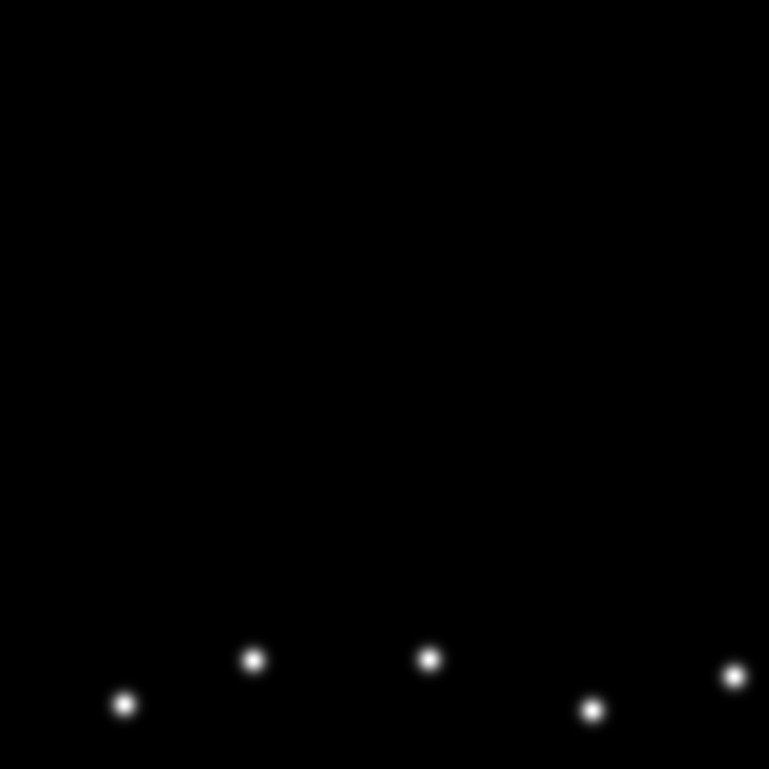

Supplement: Supplemental Information 2 [file peerj-cs-09-1451-s002.zip › ┤·┬δ/data/testdata/targets/center_target.png]

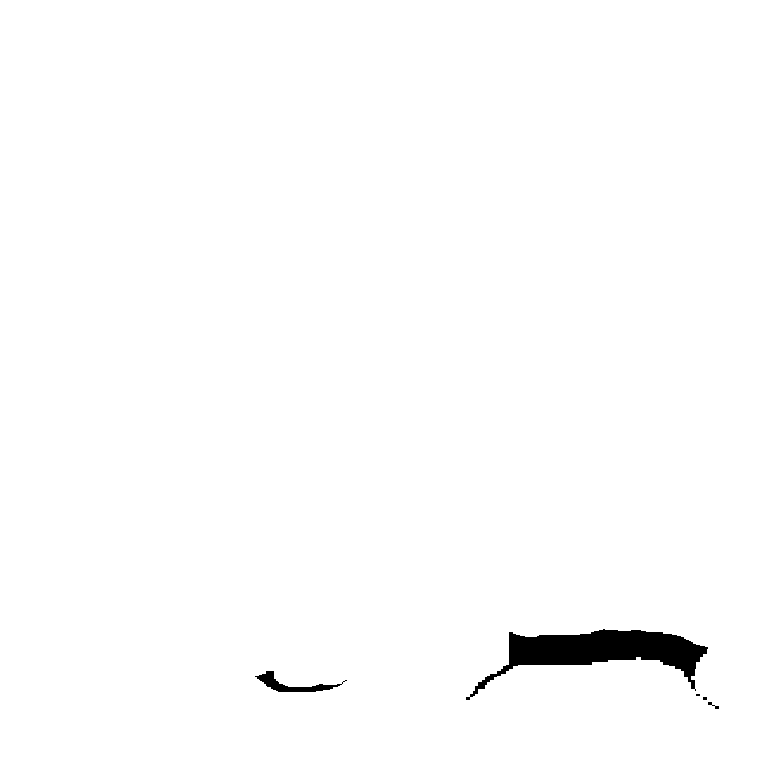

Supplement: Supplemental Information 2 [file peerj-cs-09-1451-s002.zip › ┤·┬δ/data/testdata/targets/center_weights.png]

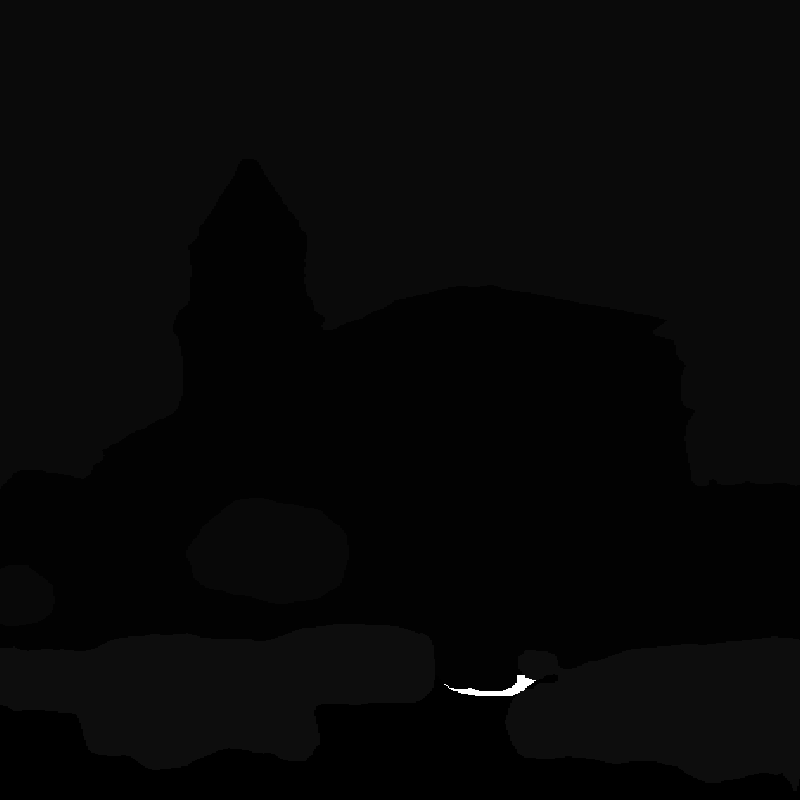

Supplement: Supplemental Information 2 [file peerj-cs-09-1451-s002.zip › ┤·┬δ/data/testdata/targets/eval_semantic_target.png]

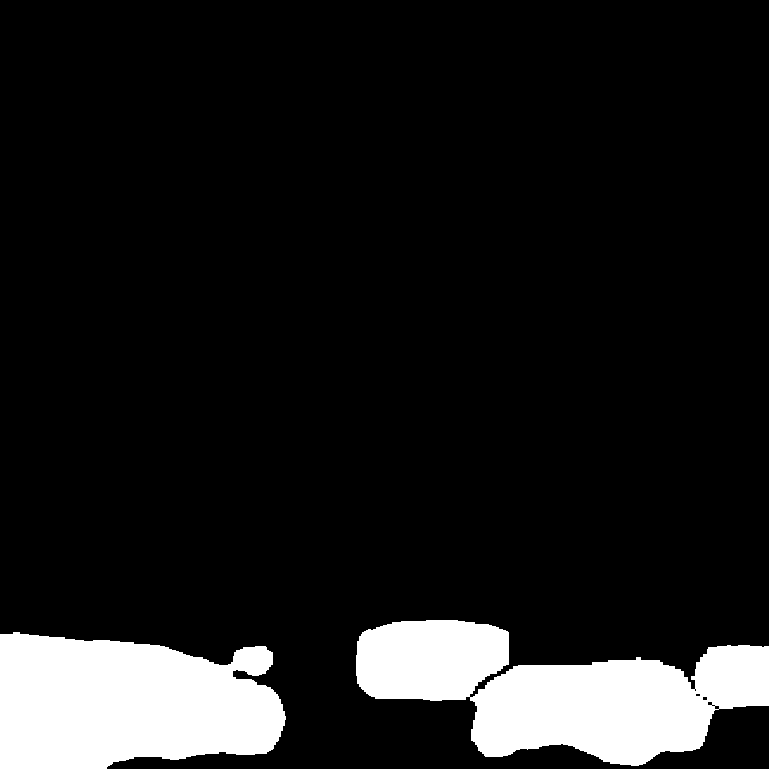

Supplement: Supplemental Information 2 [file peerj-cs-09-1451-s002.zip › ┤·┬δ/data/testdata/targets/offset_weights.png]

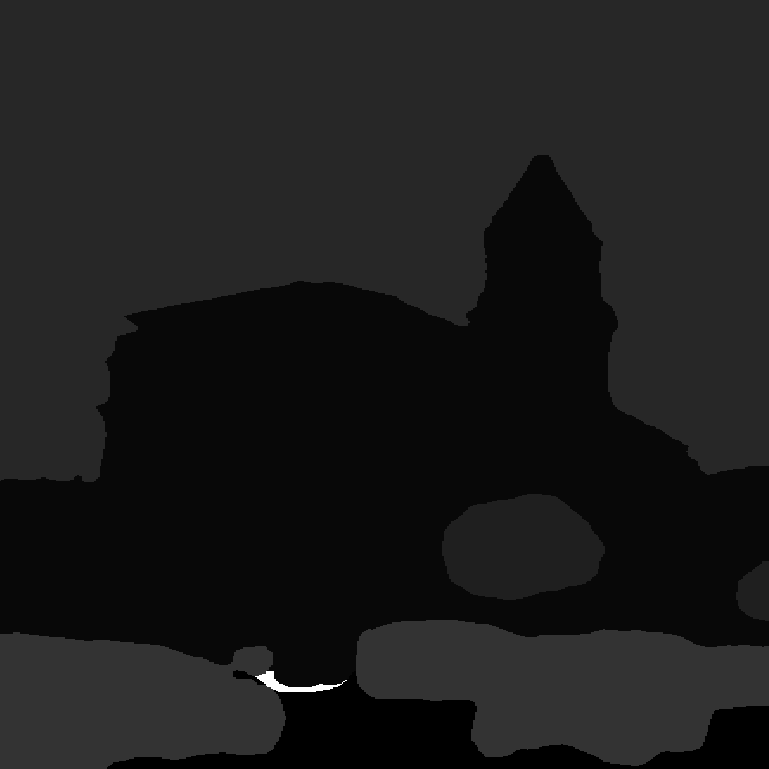

Supplement: Supplemental Information 2 [file peerj-cs-09-1451-s002.zip › ┤·┬δ/data/testdata/targets/panoptic_target.png]

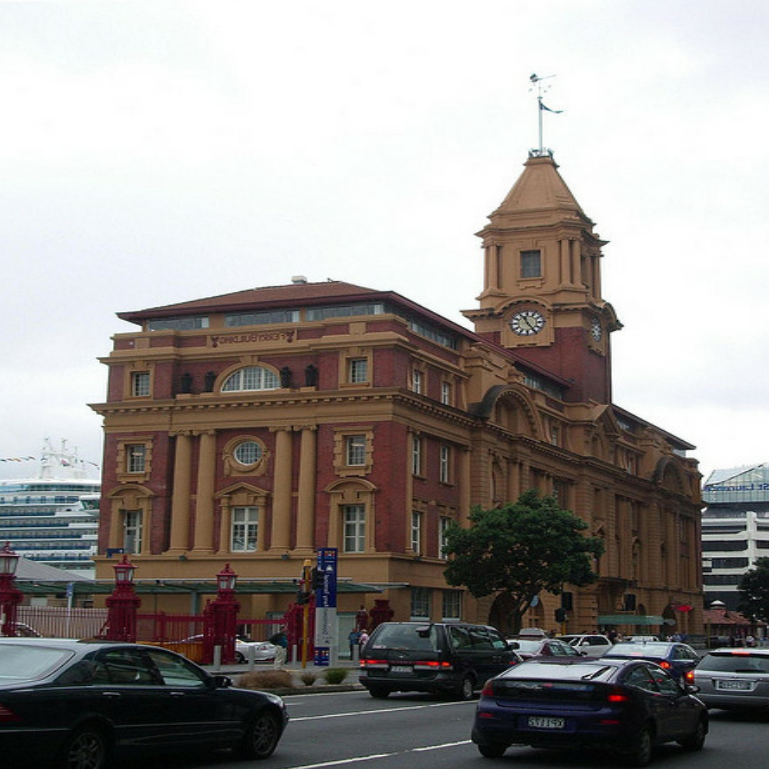

Supplement: Supplemental Information 2 [file peerj-cs-09-1451-s002.zip › ┤·┬δ/data/testdata/targets/rgb_target.png]

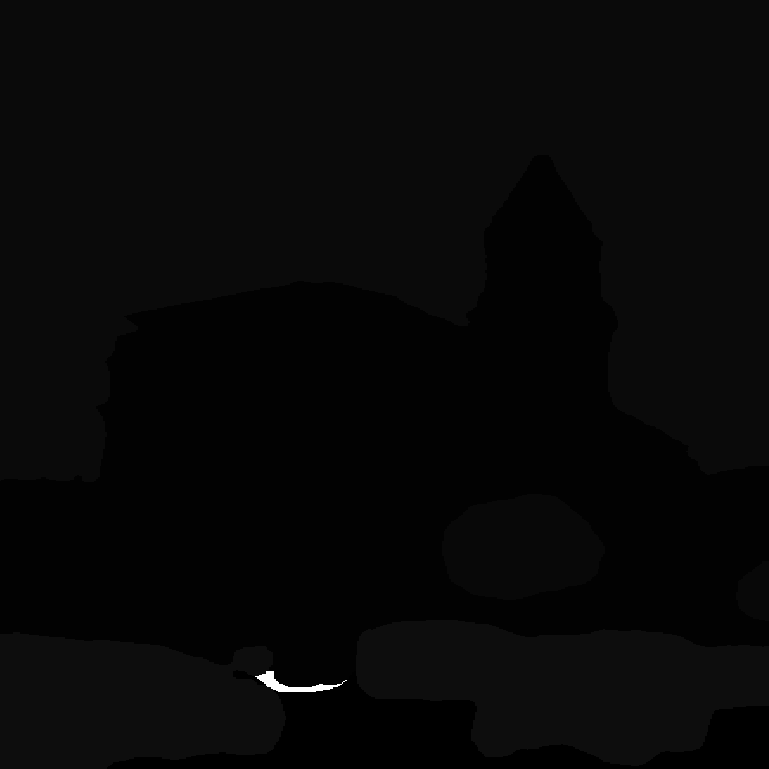

Supplement: Supplemental Information 2 [file peerj-cs-09-1451-s002.zip › ┤·┬δ/data/testdata/targets/semantic_target.png]

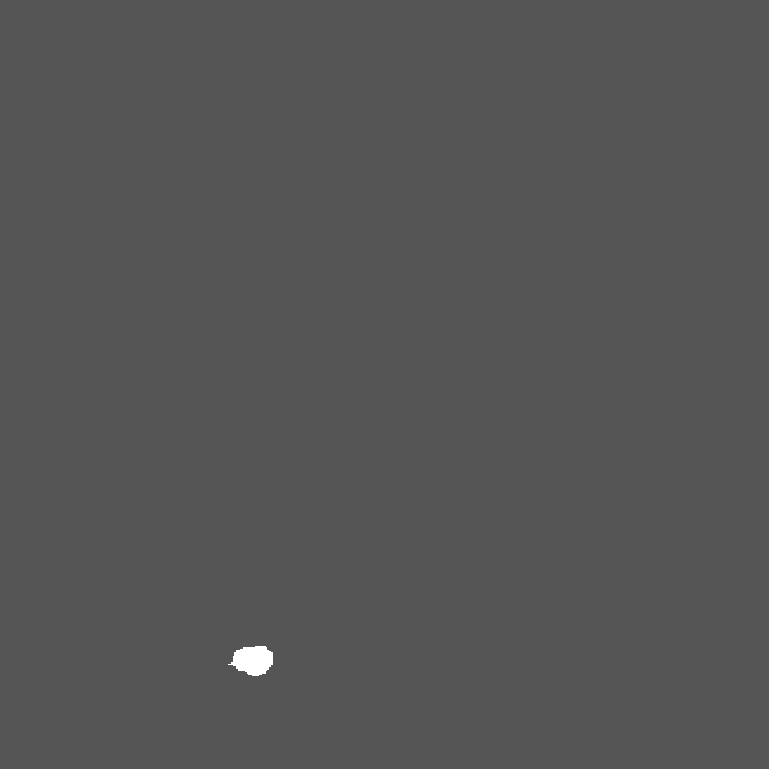

Supplement: Supplemental Information 2 [file peerj-cs-09-1451-s002.zip › ┤·┬δ/data/testdata/targets/semantic_weights.png]

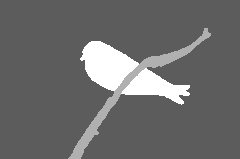

Supplement: Supplemental Information 2 [file peerj-cs-09-1451-s002.zip › ┤·┬δ/evaluation/testdata/bird_gt.png]

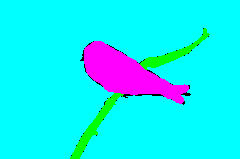

Supplement: Supplemental Information 2 [file peerj-cs-09-1451-s002.zip › ┤·┬δ/evaluation/testdata/bird_pred_class.png]

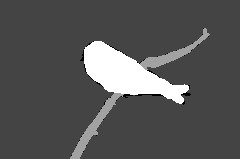

Supplement: Supplemental Information 2 [file peerj-cs-09-1451-s002.zip › ┤·┬δ/evaluation/testdata/bird_pred_instance.png]

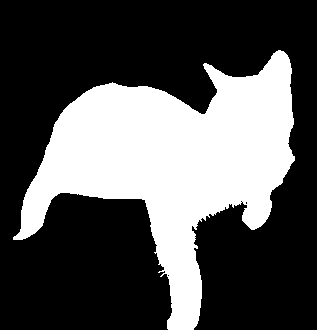

Supplement: Supplemental Information 2 [file peerj-cs-09-1451-s002.zip › ┤·┬δ/evaluation/testdata/cat_gt.png]

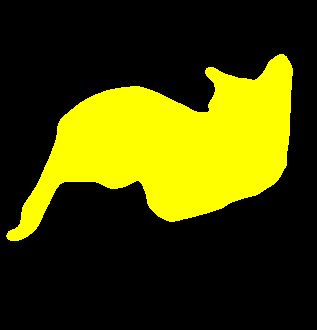

Supplement: Supplemental Information 2 [file peerj-cs-09-1451-s002.zip › ┤·┬δ/evaluation/testdata/cat_pred_class.png]

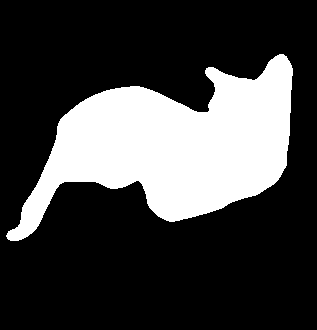

Supplement: Supplemental Information 2 [file peerj-cs-09-1451-s002.zip › ┤·┬δ/evaluation/testdata/cat_pred_instance.png]

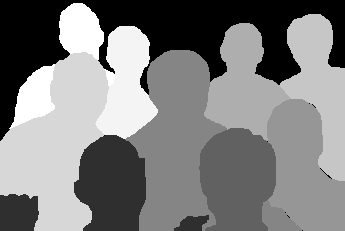

Supplement: Supplemental Information 2 [file peerj-cs-09-1451-s002.zip › ┤·┬δ/evaluation/testdata/team_gt_instance.png]

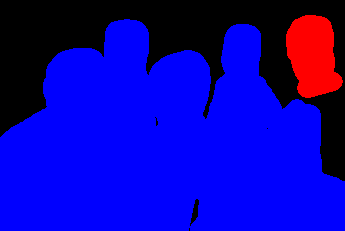

Supplement: Supplemental Information 2 [file peerj-cs-09-1451-s002.zip › ┤·┬δ/evaluation/testdata/team_pred_class.png]

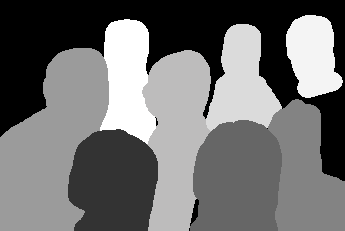

Supplement: Supplemental Information 2 [file peerj-cs-09-1451-s002.zip › ┤·┬δ/evaluation/testdata/team_pred_instance.png]
